# Training needs in telerehabilitation: results of a cross-sectional online survey with therapists and patients

**DOI:** 10.3389/fpubh.2025.1688055

**Published:** 2025-12-11

**Authors:** Anna Lea Stark-Blomeier, Stephan Krayter, Christoph Dockweiler

**Affiliations:** 1 Chair of Digital Public Health, Department of Social Sciences, Faculty of Arts and Humanities, University of Siegen, Siegen, Germany; 2 Chair of Information Systems, esp. IT for the Aging Society, School of Economic Disciplines, University of Siegen, Siegen, Germany

**Keywords:** rehabilitation, aftercare, education, needs assessment, competency, curriculum, telemedicine, telerehabilitation

## Abstract

**Introduction:**

In light of the ongoing digital transformation of healthcare, training needs analyses are essential to address the changing requirements of providing and using healthcare services. This applies in particular to the growing field of telerehabilitation. To date, a comprehensive overview of the current training landscape and potential training gaps among telerehabilitation users has been lacking. This national survey therefore aims to assess existing training opportunities and training needs of both telerehabilitation patients and therapists, taking personal and contextual differences into account.

**Methods:**

In 2023, two cross-sectional online surveys were deployed, one with telerehabilitation patients and one with telerehabilitation therapists from Germany. Drawing on the Hennessy-Hicks approach as a guiding framework, participants were asked about the relevance and their performance level of predefined telerehabilitation competencies, to determine training needs. Furthermore, data was collected on the telerehabilitation program used, the training offers received and used, and their content. Personal and contextual differences were determined using *t*-tests and *U*-tests.

**Results:**

The 262 patients and 73 therapists mostly perceived their own performance in the telerehabilitation competencies as rather good. In the past, various training offers were available. Patients were mainly instructed by their therapist or doctor and therapists via written information material in preparation for telerehabilitation. However, both target groups showed small training needs. Patients predominantly required training in self-regulatory skills, such as patience, self-awareness, and motivation, while therapists needed enhanced knowledge related to telerehabilitation and technology. Partly, patients and therapists with a low technology affinity, women as well as patients using app-based telerehabilitation showed significantly higher training needs.

**Conclusion:**

The study results can contribute to the development of a telerehabilitation curriculum for therapists and the design of needs-oriented trainings for patients. The implementation of a standardized training practice can support the competent and consistent use of telerehabilitation and contribute to improving the quality of care in the future. Further research is needed to determine which training formats and modalities are effective for the different types of competencies that are lacking.

## 1 Introduction

The remote delivery of rehabilitation services through information and communication technologies—also known as telerehabilitation—has significantly transformed the rehabilitation sector, especially during the COVID-19 pandemic (1, 2). Various digital technologies and solutions, like videoconferencing platforms, exercising apps, gaming systems or robotic devices, offer new possibilities for synchronous or asynchronous patient training at home in a variety of therapy areas including occupational therapy, speech therapy, physio- or psychotherapy (3–6).

In Germany, medical rehabilitation is an essential part of the social welfare system. It aims to enable patients to adequately cope with their illness and to participate in society. Rehabilitation aftercare serves to maintain the long-term success of rehabilitation (7). Almost one in three rehabilitation patients (approx. 30.0%) in Germany made use of aftercare in 2023 (8). The German Pension Insurance differentiates between multimodal and unimodal aftercare services. Multimodal services include therapy elements from different therapeutic areas involving several professional groups. Unimodal services consist of one therapeutic area, including training-therapeutic aftercare or aftercare for mental illnesses (7). Telerehabilitation services in Germany are mainly found in aftercare (also known as telerehabilitation aftercare). Various technology providers offer digital programs, which are part of standard care and are funded by the German Pension Insurance. The use of these programs is supervised directly by the rehabilitation facility that provided the prior treatment or by external therapists or aftercare centers. Two types of telerehabilitation programs are currently available: digital platforms that offer therapeutic content in the form of videos, images, and text, which patients can access independently on a smartphone or laptop; and videoconferencing platforms for conducting synchronous psychotherapy group sessions with a therapist, which patients can join digitally from home (9). It becomes evident that different types of programs are associated with distinct tasks and varying requirements regarding their use. A study on telerehabilitation competencies found that patients who use an app independently consider telerehabilitation and medical knowledge, motivational and self-management skills, reading/writing skills, willingness to learn, and self-efficacy expectations to be significantly more relevant than patients who use therapist-led videoconferencing for rehabilitation. Conversely, videoconference users consider empathy and teamwork to be more relevant (10). Over the past five years, the number of telerehabilitation aftercare services has increased rapidly. While less than 1.0% of all services were provided digitally in 2019, the percentage had already risen to 7.4% by 2023 (8).

As telerehabilitation becomes more widespread, it is essential to prepare patients and therapists for its effective use. Comprehensive training for digital therapy has a number of advantages. Thus, various studies show that telehealth or telerehabilitation training can reduce reservations and improve acceptance towards the tool (11–13), increase self-efficacy and confidence in usage (11, 14) as well as contribute to a better quality of care (15–17). Regarding the high potential of user training, the question arises as to how telerehabilitation patients and therapists are already trained and whether they are adequately prepared for usage. Corresponding analyses of the training landscape can be found for the broader field of telehealth and telemedicine. In a review, Edirippulige and Armfield (18) identified nine studies on telehealth education and training for practitioners. The trainings involved analog classroom lessons or eLearning and covered telehealth terminology, clinical applications, evidence and technological aspects. In their scoping review, Stovel et al. (19) found 35 articles on implemented telemedicine curricula for physicians. Delivery methods included lectures, hands-on training, directed reading, online modules, reflection, simulation and group discussions and mainly covered content about the technology, as well as clinical or communication skills. In another recent scoping review (20), 13 studies on training patients in the use of telehealth were identified. Training often took place in form of preparatory phone calls and written instructions about the technical handling of the digital tool. An overview of existing training options and training needs in telerehabilitation does not yet exist. Studies or reviews only report on missing skills (21, 22) or lacking instructions and trainings for therapists and patients (17, 23–26).

To adequately prepare users for telerehabilitation in the future, a comprehensive analysis of training needs is necessary. The use of evidence-based, standardized approaches is recommended in order to systematically identify training gaps and to develop training curricula not on the basis of assumptions, but in line with actual needs to achieve a more effective use of limited resources in education and service provision (27). One of the most frequently used, validated training needs analysis (TNA) instrument is the Hennessy-Hicks TNA questionnaire (28), which is also approved by the World Health Organization. It aims on the identification of training needs of healthcare professionals from all disciplines and covers 30 core healthcare tasks and competencies in five sub-sections, including (1) research, (2) communication/teamwork, (3) clinical tasks, (4) administration, and (5) management/supervision. Along the 30 items, a 7-point scale is used to determine how important a task is to the respondent’s job (relevance) and how well the task is currently performed (performance level). A training need exists if the relevance of a task or competency is rated higher than the perceived level of one’s own performance. The utilization of the tool facilitates the identification and prioritization of training needs at individual, group or organizational level. Consequently, it enables the development and implementation of tailored trainings (28, 29).

Against this background, this study aims on the identification of existing training offers and current training needs of therapists and patients in the context of telerehabilitation in Germany. The following research questions were addressed:

- What training offers are available for therapists and patients to prepare for telerehabilitation?
- What training needs do therapists and patients have with regard to telerehabilitation?
- How do the training needs of therapists and patients differ in terms of personal and context-related characteristics?

## 2 Materials and methods

### 2.1 Study design

We conducted two cross-sectional online surveys, one with telerehabilitation patients and one with telerehabilitation therapists from Germany. The surveys dealt with the steps and required competencies in telerehabilitation (10), and evaluated available training offers and training needs. This article only reports on the training offers and needs. We followed the STrengthening the Reporting of OBservational studies in Epidemiology (STROBE) guideline for cross-sectional studies (30) (see Supplementary File S1).

### 2.2 Study population and recruitment

The target group consisted of therapists from Germany who had been involved in the planning and delivery of a telerehabilitation program approved by the German Pension Insurance (these were all telerehabilitation aftercare services), as well as patients who had used a corresponding service within the last two years. Recruitment covered all indications and programs and aimed to achieve a complete survey. We identified a total of 351 rehabilitation facilities that offered telerehabilitation aftercare as well as twelve program providers and teletherapy clinics (see www.nachderreha.de). All of these were contacted by e-mail with the request to forward the surveys to their own therapists and patients. In addition, twelve rehabilitation-specific associations and all 377 self-help contact centers (see www.nakos.de/adressen/rot/) were approached, and posts were placed in relevant forums (two closed Facebook groups and one patient forum). The facilities and individuals were contacted in May and June 2023 and reminded several times to participate (survey period: June–August). Digital flyers and information brochures were provided. Participants were forwarded to the online survey via the link in the email and the QR code on the flyer.

### 2.3 Survey development and measures

LimeSurvey was used to develop two online surveys following the question formulation guideline by Porst (31). The final online surveys (translated from German to English, see Supplementary File S2) included closed and open questions and a total of six sections relevant for TNA. The first section covered questions about the telerehabilitation program used, including the type of program, the targeted indication group, the frequency of usage, as well as the premature termination (for patients).

Next, training needs were assessed based on the Hennessy-Hicks TNA questionnaire. Therefore, we assessed the relevance of predefined telerehabilitation competencies on a scale of 1–7 (1 = not at all important; 7 = very important), and the own performance level of these competencies on a scale of 1–7 (1 = not at all pronounced; 7 = very pronounced). The predefined competencies were not identical to the 30 items from the Hennessy-Hicks instrument, but were specific competencies relevant to telerehabilitation that were identified in a scoping review and qualitative interviews (32, 33). However, there are strong overlaps with the items from the Hennessy-Hicks instrument, e.g., in the competencies teamwork, communication, empathy, technical, analytical and adaptability skills. The relevance and performance level were thus assessed for six or, respectively, four knowledge areas, twelve skills, seven attitudes and three experiences relevant for patients and therapists using telerehabilitation.

Furthermore, we assessed the extent to which the predefined competencies were covered (C) in previous training offers on a scale of 1–7 (1 = not at all covered; 7 = completely covered). Another section examined if certain information and training options were offered to the participants and whether they made use of them. The last two sections addressed the technology affinity following Franke et al. (34) on a scale of 1–6 (a higher number indicating a higher affinity) and sociodemographic data including gender, age, highest educational/professional qualification, and for patients, access to social support regarding telerehabilitation usage.

We conducted a qualitative pretest (35) with two scientists in digital public health/telerehabilitation, one telerehabilitation therapist and two specialists from different telerehabilitation program providers. The experts tested the technical functionality, the comprehensibility of the questions, the suitability of the answer options and answer formats, and the completeness of the content (content validity). This resulted in minor adjustments to some formulations.

### 2.4 Data collection

The participants gave their consent to participate when filling out the survey. Data collection ran from June until August 2023 and was anonymous. Thus, we did not collect any information that would allow conclusions to be drawn about the identity of the participants, nor was their affiliation with any of the institutions contacted during the recruitment process recorded. 272 patients and 74 therapists filled out the questionnaire completely and 209 patients and 50 therapists incompletely (43.5% vs. 40.3% drop-out).

### 2.5 Data processing and analysis

Data were analyzed using Stata 15.1. We listwise deleted incomplete cases that gave no information on age, gender or education, as well as cases that indicated “diverse” for gender as the group size was too small for group comparison. The adjusted data set comprised 262 patients and 73 therapists. Descriptive statistics (frequencies, medians, means and standard deviations) were used to outline the characteristics of respondents and distribution of responses.

*T*-tests and *U*-tests were used to examine if there were significant differences regarding training needs between subgroups. The dependent variable was the training need for a competency or competency dimension. Therefore, the assessed competencies were combined into index variables for each competency dimension (knowledge index, skill index, attitude index, experience index). A new interval-scaled variable was created (scale −6 to 6) as the difference (D) between the perceived performance level (L) and relevance (R) of a competency. If this value is negative, there is a need for training. The bigger this difference, the greater the training need. When interpreting the training needs, the perceived relevance of the competency is considerable, as training needs for less relevant competencies (< 4) can be classified as less urgent (low intervention priority). Training needs are more urgent (high intervention priority) when the relevance of a competency is high (≥ 4) and the performance level is low (< 4) (28). The independent group variables had a nominal scale or were converted to a nominal scale with two independent groups. This included gender (male vs. female), program used (independent app training vs. therapist-led videoconferencing), therapist’s job (teletherapist only vs. also working on-site/hybrid), age (young vs. old) and technology affinity (low vs. high). Regarding age, the patient sample was divided into *<*50 and ≥50 years along the average age of German rehabilitants of 53 years (36). This also corresponds to the median age of the sample (“50 to 59 years”). Since there are no statistics on the average age across all therapist groups in Germany, the therapist sample was split into *<*40 and ≥40 years according to the median age of the sample, as commonly practiced in research (median split) (37, 38). At the same time, this cutoff is supported by statistics on individual groups of therapists, according to which psychological psychotherapists, physical therapists, occupational therapists, and speech therapists are located in the median in the age group “40 to 49 years” (39). Technology affinity was divided into “low” (1–3.5 points) and “high” (3.6–6 points). Supplementary File S3 describes the group variables and sizes.

We then checked the prerequisites for *t*-tests [normal distribution and equal variances in both groups, Schober and Vetter (40)] using Shapiro–Wilk and Levene tests (see Supplementary File S3). Since *t*-tests are comparisons of means, we may expect that with large sample sizes (*>*30 per group) the assumption of normally distributed data is no longer critical [30], for smaller groups *U*-tests must be carried out. Welch-tests must be performed in case of normal distribution but no variance homogeneity (41).

## 3 Results

### 3.1 Sample description

The adjusted data set comprised 262 patients and 73 therapists (see Table 1). The median age of patients was between 50 and 59 years, the median age of therapists between 40 and 49 years. Both samples were mainly female (76.3 and 67.1%) and slightly more participants had a high affinity for technology (50.8 and 60.3%). The patient sample had a medium level of education, with more than half of the participants holding a vocational or university qualification. The therapist sample had a high level of education, with more than half of the participants holding a master’s or a doctoral degree. Most of the therapists worked in sports/movement therapy (54.8%), followed by physiotherapy (39.7%) and psychotherapy (37.0%). Around 20.0% worked purely as teletherapists and around 80.0% also worked on-site (hybrid).

**Table 1:** Characteristics of the sample, absolute/relative frequencies (n_patients_ = 262, n_therapists_ = 73).

Variable	Value	Patients		Therapists	
		*n*	%	*n*	%
Age	20–29 years	2	0.8	15	20.5
	30–39 years	23	8.8	20	27.4
	40–49 years	45	17.2	17	23.3^a^
	50-59 years	141	53.8^a^	14	19.2
	60-69 years	51	19.5	7	9.6
	*N*	262	100.0	73	100.0
Gender	Male	62	23.7	24	32.9
	Female	200	76.3	49	67.1
	*N*	262	100.0	73	100.0
Qualification	No educational qualifications	1	0.4	0	0.0
	“Hauptschulabschluss” (lower secondary school qualification)	5	1.9	0	0.0
	“Mittlere Reife” (secondary school certificate)	46	17.6	0	0.0
	“Fachhochschulreife” (specialized A-levels)	20	7.6	2	2.7
	“Allgemeine Hochschulreife” (A-levels)	26	9.9	3	4.1
	Vocational training	87	33.2	12	16.4
	Bachelor’s degree / equivalent educational program	30	11.5	12	16.4
	Master’s degree / equivalent educational program	43	16.4	37	50.7
	Doctoral degree	4	1.5	7	9.6
	*N*	262	100.0	73	100.0
Technology affinity	Low (1–3.5 points)	129	49.2	29	39.7
	High (3.6–6 points)	133	50.8	44	60.3
	*N*	262	100.0	73	100.0
Type of therapy^*^	Psychotherapy	x	x	27	37.0
	Physiotherapy	x	x	29	39.7
	Sports/movement therapy	x	x	40	54.8
	Speech therapy	x	x	2	2.7
	Occupational therapy	x	x	9	12.3
	Other	x	x	6	8.2
	*N*	x	x	113	154.7
Type of job	Only teletherapist	x	x	15	20.5
	Teletherapist and on-site therapist (hybrid)	x	x	58	79.5
	*N*	x	x	73	100.0

^*^Multiple answers possible. Therefore, the total number exceeds 73.

a This age group represents the median age.

Both patients and therapists more frequently used or supervised a telerehabilitation app where patients trained independently (56.9 and 79.5%). Less frequently, they used or provided a therapist-led videoconference (43.1 and 20.5%). The majority of patients used the program for psychosomatic indications (64.9%), while therapists mainly used it in the context of orthopedics (52.1%). The average frequency of usage (median) was once a week for patients and several times a week for therapists. Of the 45 patients who no longer used the program at the time of the survey, ten had terminated the program prematurely (six due to usage difficulties). More than half of the patients (56.5%) reported that they had no access to people who could support them in the usage of the program (see Supplementary File S4).

### 3.2 Availability, usage and content of training in telerehabilitation

The survey shows that almost all telerehabilitation users received (98.1% patients, 95.9% therapists) and used (97.0% patients, 95.9% therapists) at least one training opportunity in preparation or support. Regarding the training format and modality, the majority of patients (82.8%) had an individual conversation with their therapist or doctor in preparation for telerehabilitation (see Supplementary File S5). Around three out of five patients (60.7%) used consulting services (e.g., by telephone, email or chat) and slightly less than half used written information material (46.2%) or information videos (45.8%). More than one in five stated that on-site workshops with the opportunity to try out the program (22.1%) and interactive online webinars (21.8%) were not offered, but that they would have liked to attend them. In the case of information videos (16.4%), online presentations (16.4%) and on-site presentations at the rehabilitation facilities (15.3%), around one in six stated that these were not offered, but that they would have liked to attend them. However, around half of the patients declared that online presentations (57.6%), online webinars (56.9%) and on-site workshops (49.6%) were not offered, but not necessary either.

For therapists, written information material was the most frequently used option to prepare for telerehabilitation (80.8%). Consulting services (e.g., by telephone, email or chat) were used by more than two thirds (69.9%), information videos (60.3%), interactive online webinars (53.4%), on-site presentations (52.1%) and on-site workshops with the opportunity to try out the program (50.7%) by more than half. Vocational or university lectures as well as certified trainings in telemedicine or telerehabilitation were offered the least frequently (19.2 and 28.8%, regardless of actual use). In the case of certified trainings, approximately 30.0% reported that no such offer was available, although they would have welcomed it. Around 40.0% also had no access to such an offer but reported that they did not need it. For vocational or university lectures, about 20.0% lacked access but would have appreciated such an offer, whereas about 60.0% had no such offer and expressed no need for it. Table 2 shows the three most frequently used training offers for both target groups.

**Table 2:** Most frequently used training offers for telerehabilitation, absolute/relative frequencies (n_patients_ = 262, n_therapists_ = 73).

Training offer used by patients	*n* (%)	Training offer used by therapists	*n* (%)
Conversation with therapist/doctor	217 (82.8)	Written information material	59 (80.8)
Consulting services (e.g., by telephone, email, chat)	159 (60.7)	Consulting services (e.g., by telephone, email, chat)	51 (69.9)
Written information material	121 (46.2)	Information videos	44 (60.3)

^*^Multiple answers possible. See Supplementary File S5 for all data regarding training usage.

Furthermore, it was examined to which extent the competencies relevant for telerehabilitation were covered (C) in existing trainings or rather what content the trainings included (see Supplementary File S5). We assessed the coverage in existing trainings for the knowledge areas, skills and attitudes, but not for the prior experiences as these are often informally acquired and are generally not systematically covered in formal trainings. For patients, the average coverage of competencies in previous telerehabilitation trainings was 4 points or more for most competencies (19/23, 82.6%). On a scale of 1 “not at all covered” to 7 “completely covered,” this means that these competencies were at least partially trained or addressed in the training offers. Telerehabilitation knowledge (C = 5.1), self-interest in the program (C = 5.0) and self-awareness (C = 4.8) were addressed most extensively in patient training. Technology affinity (C = 3.4), technology acceptance (C = 3.6), technology skills (C = 3.8), and reading/writing skills (C = 3.9) were rather not addressed. For therapists, the average coverage of competencies in previous telerehabilitation trainings was less than 4 points for most competencies (19/25, 76.0%). This means that these competencies were rather not trained or addressed in the training offers. Telerehabilitation knowledge (C = 5.4) and implementation knowledge (C = 4.7) were addressed most extensively in therapist training. Frustration tolerance (C = 3.1), self-awareness (C = 3.1) and patience (C = 3.2) were addressed the least extensively.

### 3.3 Performance and training needs in telerehabilitation

Patients and therapists were asked to rate their own performance level (L) for the competencies relevant to telerehabilitation on a scale of 1 “not at all pronounced” to 7 “very pronounced” (see Supplementary File S6). The performance level of therapists was on average 4 points or more for all competencies, indicating that telerehabilitation competencies were at least slightly present. For patients, the performance level was below 4 points for only one competency, all other telerehabilitation competencies were at least slightly present. The most developed competencies among patients were reading/writing skills (L = 6.3), self-interest in the telerehabilitation program (L = 6.2) and open-mindedness (L = 5.8), among therapists therapeutic-professional skills (L = 6.2), self-management (L = 6.1) and empathy (L = 6.1). The least developed competencies among patients were experience with health apps (L = 3.7), experience with analog therapy (L = 4.5) and patience (L = 4.6), among therapists technology knowledge (L = 4.4), process knowledge (L = 4.4) and technology skills (L = 4.8).

The training needs were calculated as the difference (D) between the relevance and performance level of a competency or competency dimension (see Supplementary File S6). Patients showed a small need for training in 17 of the 26 telerehabilitation competencies (65.4%). When looking at the competency dimensions (indices), the greatest need for training was in the knowledge areas (D = −0.33) and skills (D = −0.20). For the experience index and the individual relevant experiences, the perceived performance level was on average higher than the perceived relevance, indicating no overall training need. The greatest training needs for patients regarding the individual competencies lay in patience (D = −0.94), self-awareness (D = −0.78) and motivational skills (D = −0.69). This is especially notable as these were rated as highly relevant by the patients (R ≥ 5.5). In fact, self-awareness was the second most relevant telerehabilitation competency (R = 5.9). For the most and the third most relevant competencies “self-interest in the program” and “self-management,” the need for training was rather small (D = −0.13 and −0.33). The three greatest training needs of all patients mentioned are identical to the subgroups of patients who used therapist-led videoconferencing or independent app-based training (see Table 3).

**Table 3:** Highest telerehabilitation training needs by patients, means.

Rank	Patients (all, *n* = 262)	Patients (video user, *n* = 113)	Patients (app user, *n* = 149)
1	Patience (*M* = −0.94)	Self-awareness (*M* = −0.97)	Patience (*M* = −0.95)
2	Self-awareness (*M* = −0.78)	Patience (*M* = −0.93)	Motivational skills (*M* = −0.74)
3	Motivational skills (*M* = −0.69)	Motivational skills (*M* = −0.62)	Self-awareness (*M* = −0.64)

^*^See Supplementary File S6 for all data regarding training needs.

Therapists showed a small need for training in ten of the 28 telerehabilitation competencies (35.7%). When looking at the competency dimensions (indices), the only need for training was in the knowledge areas (D = −0.23). For the experience index and the individual relevant experiences, the perceived performance level was on average higher than the perceived relevance, indicating no overall training need. Only three of the attitudes (technology acceptance, open-mindedness and frustration tolerance) and two of the skills (therapeutic-professional and motivational skills) showed a small need for training. Overall, the greatest training need for therapists regarding the individual competencies lay in telerehabilitation knowledge (D = −0.41), technology knowledge (D = −0.33) as well as legal and implementation knowledge (D = −0.32). The training need for telerehabilitation knowledge is particularly noteworthy, as this was the third most relevant telerehabilitation competency for therapists (R = 6.1). For the most and second most relevant competencies “therapeutic-professional skills” and “medical knowledge,” the need for training was rather small (D = −0.15 and −0.04). The three greatest training needs of all therapists mentioned above differ in part from those of the therapist subgroups. While the greatest training needs of the app-using and on-site / hybrid therapists were also in the knowledge areas mentioned, therapists who guide a videoconference had the greatest training needs in frustration tolerance (D = −0.67) and therapists who work as teletherapists only in communication and motivational skills (D = −0.47), technology acceptance and experience with analog therapy (D = −0.33) in addition to knowledge (see Table 4).

**Table 4:** Highest telerehabilitation training needs by therapists, means.

Rank	Therapists (all, *n* = 73)	Therapists (video user, *n* = 15)	Therapists (app user, *n* = 58)	Therapists (teletherapist, *n* = 15)	Therapists (on-site, *n* = 58)
1	Telerehabilitation knowledge (*M* = −0.41)	Frustration tolerance (*M* = −0.67)	Telerehabilitation knowledge (*M* = −0.41)	Communication/Motivation (*M* = −0.47)	Telerehabilitation knowledge (*M* = −0.48)
2	Technology knowledge (*M* = −0.33)	Technology knowledge (*M* = −0.60)	Legal knowledge (*M* = −0.36)	Technology acceptance/Experience in analog therapy (*M* = −0.33)	Technology/Implemen-tation knowledge (*M* = −0.38)
3	Legal/Implementation knowledge (*M* = −0.32)	Telerehabilitation knowledge (*M* = −0.40)	Implementation knowledge (*M* = −0.33)	Empathy/Legal knowledge (*M* = −0.20)	Legal knowledge (*M* = −0.34)

^*^Although the groups of videoconference users and teletherapists as well as app users and on-site therapists are the same size, the respective groups are not composed of identical therapists. See Supplementary File S6 for all data regarding training needs.

**Figure 1:**
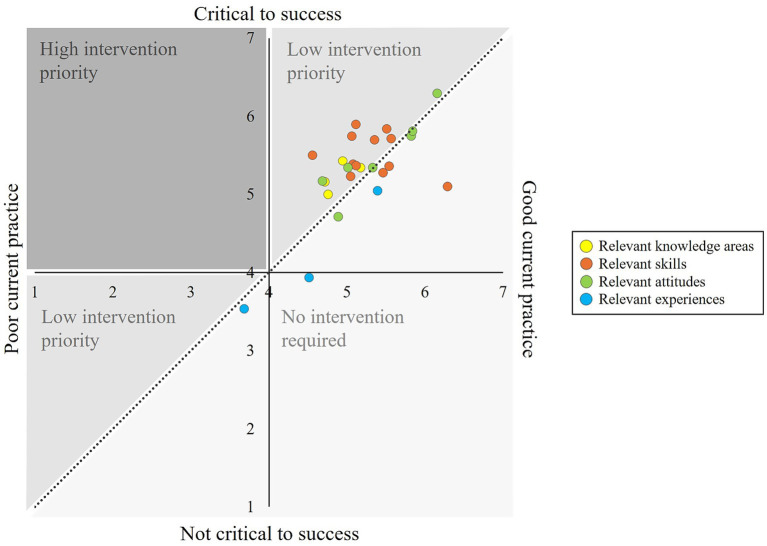
Training needs of patients regarding telerehabilitation usage.

**Figure 2:**
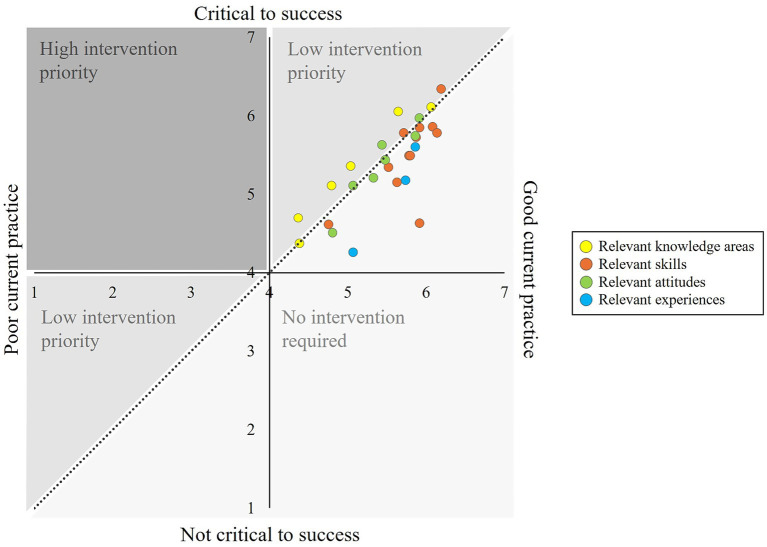
Training needs of therapists regarding telerehabilitation usage.

Figures 1, 2 visualize the telerehabilitation training needs of patients and therapists. The Y-axis shows the relevance of a competency from 1 to 7. If a competency is classified as highly relevant or “critical to success,” it is at the top of the diagram. The X-axis shows the performance level of a competency from 1–7. If a competency is highly developed or has a “good current practice,” it is located further to the right in the diagram. If a competency lays above the diagonal (relevance is higher than performance level), there is a need for training. The data points stand for the individual telerehabilitation competencies, with yellow dots representing the four knowledge areas relevant for patients or the six knowledge areas relevant for therapists, orange dots representing the twelve skills relevant for patients and therapists, green dots representing the seven attitudes relevant for patients and therapists and blue dots representing the three experiences relevant for patients and therapists. The bigger the gap between relevance and performance, the further away the data point is from the linear and the greater the training need. In comparison between patients and therapists, patients had higher training needs across all competency dimensions (indices). They also showed a need for training in more individual competencies. Only in four competencies (telerehabilitation and legal knowledge, technology acceptance, open-mindedness) was the need for training on average slightly higher for therapists. The figures show that the greatest telerehabilitation training needs for patients lay in individual skills (orange dots) and for therapists in individual knowledge areas (yellow dots). Overall, all identified training needs can be classified as less urgent, as there were only small differences between relevance and performance level, and the corresponding competencies were already well developed by patients and therapists (L ≥ 4).

### 3.4 Group differences in training needs

The subgroup analysis showed significant group differences in training needs for telerehabilitation. For better readability, only those competencies are reported below where significant differences were found between subgroups and where at least one of the two subgroups showed an absolute need for training (D < 0). All other results and *p*-values can be found in Supplementary File S7.

With regard to the competency dimensions (indices), patients with a low affinity for technology showed a significantly higher need for training in knowledge areas and attitudes than patients with a high affinity for technology. Regarding attitudes, patients with a high affinity showed no absolute training need. Furthermore, women had a significantly higher need for training with regard to knowledge areas and attitudes compared to men, who showed no need for training here. Next, patients who independently used an app for telerehabilitation showed significantly higher training needs in relevant attitudes than patients using a guided videoconference who showed no training need at all. When looking at the individual competencies, the most significant differences can be seen regarding technology affinity. Patients with a low affinity for technology showed a significantly higher need for training for over half of the telerehabilitation competencies (53.8%), including legal knowledge, technology knowledge, technology skills, adaptability skills, self-management, technology affinity, technology acceptance, willingness to learn, open-mindedness, frustration tolerance, self-efficacy expectation, self-interest in the program, experience with digital health apps and experience with digital tools. Of these competencies, patients with a high affinity for technology only showed a need for training in adaptability skills, self-management and frustration tolerance. Regarding the telerehabilitation program used, patients independently using a telerehabilitation app showed a significant higher need for training for technology affinity, willingness to learn, open-mindedness and self-interest in the program compared to patients using videoconferences who had no training needs. Next, patients using a videoconference for telerehabilitation showed a significant higher need for training in teamwork, compared to patients training independently with an app who showed no training need here. Women showed a significant higher training need in legal knowledge, technology knowledge, technology skills, technology affinity and technology acceptance compared to men who had no training needs here. Men showed a significant higher training need for empathy, compared to women who showed no training need. Finally, older patients had a significantly higher training need in patience compared to younger patients.

With regard to the competency dimensions (indices), therapists with a low technology affinity showed a significant higher training need in knowledge areas and attitudes, compared to therapists with a high technology affinity who showed no training need here (D ≥ 0). Women had a significantly higher need for training with regard to knowledge areas and attitudes, compared to men who showed no training needs. When looking at the individual competencies, the most significant differences can be seen regarding technology affinity. Therapists with a low affinity for technology showed a significantly higher need for training for more than a third of the competencies (35.7%), including telerehabilitation knowledge, technology knowledge, medical knowledge, implementation knowledge, process knowledge, technology skills, technology affinity and frustration tolerance. Of the competencies mentioned, therapists with a high affinity for technology only showed a need for training in telerehabilitation knowledge. For process knowledge, technology skills and technology affinity women showed a significant higher training need compared to men who showed no training need. Furthermore, teletherapists had a significant higher training need in communication skills and motivation skills, compared to hybrid therapists who showed no training need here. As for previous experience in analog therapy, younger therapists showed a significantly higher need for training compared to older therapists, who showed no need for training. Lastly, therapists leading a videoconference program had a significant higher training need for frustration tolerance compared to therapists supervising an app-based program who showed no training need.

Tables 5, 6 summarize the number of competencies for which a certain subgroup characteristic is associated with a significantly higher need for training. Only those competencies are counted for which an absolute need for training (D < 0) was determined for at least one of the subgroups.

**Table 5:** Number of competencies with significant higher training needs by patient subgroups.

Subgroup 1	*n*	Subgroup 2	*n*
Patients with low technology affinity^a^	14	Patients with high technology affinity^a^	0
Male patients	1	Female patients	5
Patients using videoconferencing programs	1	Patients using app-based programs	4
Young patients^b^	0	Old patients^b^	1

a Technology affinity was divided into low (1–3.5 points) and high (3.6–6 points) on a scale from 1–6.

b Patients’ age was divided into young (under 50 years) and old (50 years or older).

**Table 6:** Number of competencies with significant higher training needs by therapist subgroups.

Subgroup 1	*n*	Subgroup 2	*n*
Therapists with low technology affinity^a^	8	Therapists with high technology affinity^a^	0
Male therapists	0	Female therapists	3
Therapists supervising videoconferencing programs	1	Therapists supervising app-based programs	0
Young therapists^b^	1	Old therapists^b^	0
Only teletherapist	2	On-site and teletherapist (hybrid)	0

a Technology affinity was divided into low (1–3.5 points) and high (3.6–6 points) on a scale from 1–6.

b Therapists’ age was divided into young (under 40 years) and old (40 years or older).

## 4 Discussion

### 4.1 Main findings and comparisons with prior work

In two online surveys, patients and therapists from Germany were asked about their experiences with telerehabilitation. The results show that various offers were available for their preparation and support. They often used consulting services via telephone, email, or chat as well as written information material. Patients were instructed by their therapist or doctor and therapists via information videos. With regard to the competencies relevant to telerehabilitation—including the dimensions knowledge, skills, attitudes and previous experience—both target groups already had a good level of competency for the most part, meaning that existing training needs were classified as less urgent. Nevertheless, patients showed small training needs for about two-thirds and therapists for about one-third of the telerehabilitation competencies. When looking at the competency dimensions, the training need in the knowledge dimension was highest for both target groups. However, among patients, several individual skills showed the greatest need for training. The most significant differences in training needs were found in the users’ affinity for technology. Partly, women had higher training needs in both target groups as well as patients using app-based telerehabilitation.

As described before, training needs in telerehabilitation have not yet been systematically and comprehensively accessed. Initial findings are evident from a mixed-methods study of a telerehabilitation program for patients with musculoskeletal or oncological diseases in Austria including synchronous videoconferencing (42). In focus groups, the therapists pointed out that besides technical training for handling the program they have a training need for didactic competencies (e.g., transferring rehabilitation strategies to the online setting and communicating via digital tools). For patients, the interviewed therapists identified further training needs in technical and communication skills (42). This is similar to our study, as we found that therapists who use video-assisted telerehabilitation not only need training in the use of the technology or program, but also have smaller training needs in didactic competencies such as adaptability, communication and motivation skills. With regard to patients who use videoconferencing for telerehabilitation, we found that although they have training needs related to technical use and communication skills, these were rather small.

Further comparisons can be made with studies from the broader field of telehealth. In our study, we found that telerehabilitation therapists had the greatest training needs in knowledge about telerehabilitation (e.g., the programs’ content and steps), technology (e.g., how to install and troubleshoot the program), legal issues (e.g., data protection) and implementation (e.g., guidelines and procedures). Telerehabilitation patients, by contrast, showed the greatest need for training in patience, self-awareness and motivational skills. Also, Banbury et al. (43) found in their qualitative study that staff from residential aged care facilities in Australia using telehealth requires training in technical knowledge and skills, knowledge on how to implement telehealth, evidence-based information on using telehealth, as well as knowledge on telehealth policy and legal issues. Another focus group study from Norway (44) stated that healthcare professionals working in home healthcare services with no prior telehealth experience need training in implementation processes, including not only technical but communications skills and techniques appropriate for digital care. For patients, there is a lack of evidence on training needs in the overarching field of telehealth or telemedicine. The greatest barriers or challenges for patients in the use of telemedicine or telerehabilitation identified in other studies—namely problems in technical handling and missing digital skills (45–47)—do not coincide with the greatest training needs identified by us. Thus, we showed that patients need especially training in patience, self-awareness and motivational skills, which can be rather assigned to meta-cognitive or self-regulatory skills that are not directly related to the use of the technology. The fact that our patient sample showed no or only minimal need for training in technology skills, acceptance and affinity may be due to the fact that the rehabilitation facilities or program providers of the telerehabilitation programs approved by the German Pension Insurance in Germany are obliged to instruct patients in the digital program (9). Thus, our study results show that the majority of patients have already received trainings or introductions.

In our study, we identified that telerehabilitation users with a low technology affinity have more and significantly higher training needs than telerehabilitation users with a high technology affinity, whereas the users’ age tends to have no effect. According to current research, both affinity for technology and age may have an influence on the adoption, usage and successful implementation of telemedicine or telehealth services (48–52). There may be various reasons why older users in our study had (almost) no greater need for training or in other words were not dependent on more intensive preparation and support. First, it should be noted that our samples only had a limited age range. Thus, there were no very young (<20 years) and no very old participants (≥70 years) among either the patients or the therapists. In addition, the existing age groups were unevenly distributed. For example, patients who were assigned a younger age were mostly 40 years or older, and patients who were assigned an older age were mostly under 60 years old. Due to the low age variance of the samples, a potential effect of age on training needs may not have been measurable. It is also possible that age only had an indirect effect on usage or the need for training and that other variables, such as eHealth literacy, mediate the effect (53).

Another interesting finding are the differences in training needs based on gender. Thus, female patients and therapists partly showed significantly higher training needs. According to current evidence, there are inconsistent findings regarding the relationship between gender and the (successful) use of digital health services. While some systematic reviews find that women use such services significantly more often than men (54, 55), another review points out that there is currently no clear evidence of gender-specific differences in effectiveness of usage (56). However, it has been confirmed that there are structural and personal barriers for women in the use of health technologies, such as gender inequality or lack of technical skills (57). These should be taken into account when designing and implementing information and training programs for patients regarding telerehabilitation.

When interpreting the results, it should also be noted that we only surveyed patients and therapists who had already opted for telerehabilitation in the past and had experience with it. On the one hand, it may be that the patients surveyed opted for telerehabilitation because they considered their competencies sufficient for its usage. On the other hand, it is possible that rehabilitation patients who have no previous experience with telerehabilitation or who have actively decided against it may have greater telerehabilitation training needs as was assessed in our study of patients with usage experience. The group of users and non-users could therefore differ significantly in terms of their competencies. One possible reason for not using telerehabilitation could be a lack of technical skills, as some reviews indicate (4, 58). A German study shows that the main reason patients opt for conventional rehabilitation aftercare is low outcome expectations, meaning it is relevant whether they believe the digital program will achieve the desired results (59). However, further research is needed regarding acceptance and adoption factors in telerehabilitation. In the future, it will be necessary to investigate whether informing and training all rehabilitation patients in the use of telerehabilitation could increase acceptance and ultimately success in usage.

### 4.2 Implications and future directions

We found that almost all telerehabilitation users (≥ 95.0%) received at least one training opportunity. This is higher than data from another study focusing on telehealth providers, including physicians, therapists and nurses from the United States, which found a training rate of 71.4% (60). A comparative study on training rates in patients is not known to the authors. The high training rates in our study underline the fact that the requirements for rehabilitation facilities and telerehabilitation providers described in a German Pension Insurance requirements paper (9)—that is, introducing patients to the telerehabilitation program and ensuring further training for therapists in the use of the digital tools—are actually being applied in practice. It should be noted here that the requirements so far only apply to facilities and providers that implement telerehabilitation aftercare integrated into standard care financed by the German Pension Insurance. In future, it would be desirable for training requirements to also apply to telerehabilitation in outpatient or inpatient rehabilitation outside of aftercare and outside of standard care, including in-house telerehabilitative services provided by rehabilitation facilities or services provided by teleclinics that are paid for privately. Furthermore, in future, suitable training content and effective training formats should be specified in requirements papers in line with the needs and preferences of users. In our study, patients were primarily informed about telerehabilitation through conversations with their therapist or doctor and therapists through written information material. In comparison, reviews on telehealth training report that providers are primarily trained via online platforms (18) and patients via one-on-one phone calls (20), whereas telemedicine curricula for physicians primarily include lectures and hands-on training (19). There is a need for further research to identify which training formats and modalities are particularly effective in promoting the successful use of telerehabilitation and supporting its efficient integration into clinical processes. Thus, previous studies have mainly evaluated the satisfaction of training participants and not its effectiveness or impacts (18, 61).

As described before, the use of evidence-based and standardized approaches, such as the Hennessy-Hicks instrument, is recommended and essential for identifying training gaps and developing appropriate curricula (27). Current evidence shows that telemedicine or telehealth curricula for healthcare providers are neither standardized nor evidence-based. A review of telehealth patient training found that of 13 included studies, only seven reported how the training activities were developed and only two used a theory-based approach (20). Our study provides a basis for the evidence-based and standardized development of patient training or comprehensive curricula for healthcare providers in telerehabilitation. In this context, it is particularly important to focus on the telerehabilitation competencies with the greatest need for training and to examine which formats and content are suitable to build on these competencies. For example, the competencies with the greatest training needs in patients, namely self-awareness, patience and motivational skills, can be classified as meta-cognitive or self-regulatory skills (62). To promote these skills specific meta-cognitive interventions are required, that actively involve learners in planning, control, monitoring, and reflection processes. A meta-analysis in the school context shows, for example, that such interventions have a long-term impact on self-efficacy outcomes (63). When designing and implementing training offers in telerehabilitation, it will also be important in future to consider the person- and context-specific differences in training needs that we have identified. For example, users with low technical affinity showed more and significantly greater training needs than users with high technical affinity. Therapists who lead telerehabilitation videoconferences or work exclusively as teletherapists also have different training needs than therapists who supervise app-based training or work in a hybrid manner. Furthermore, we found that telerehabilitation competencies were partly insufficiently covered in existing patient trainings and were mostly inadequately addressed in existing therapist trainings. It is particularly critical that there was a need for training in the overall patient sample or one of the subgroups for the competencies that were least addressed in patient training, namely technology skills, affinity and acceptance. Among therapists, frustration tolerance is the competency least covered in trainings, although therapists who offer video-supported telerehabilitation have the greatest need for training in this competency. This highlights the need to revise existing training offers in telerehabilitation based on the study results and thus adapt them to the actual needs of users.

### 4.3 Strengths and limitations

The study was guided by the Hennessy-Hicks instrument, which is an established, globally utilized approach for assessing training needs in the healthcare sector. It is a reliable and valid instrument that is psychometrically robust even when items are replaced or added (28, 29). However, it should be mentioned here that although the telerehabilitation competencies we assessed were similar to the healthcare tasks in the instrument, we did not use the same content or wording of items, which limits the quality of our survey. In contrast to the Hennessy-Hicks approach, we have interpreted competencies that were less pronounced than relevant but had a rather good level of competency (L ≥ 4) in such a way that there was a less urgent training need. Hennessy and Hicks (28), on the other hand, see no need for training here, as along their interpretation the important task is already well performed. Although the content validity of our questionnaire was ensured through expert consultations and pretests, it should be noted that no formal psychometric validation was carried out. A further limitation is that the surveys included self-reports. It is possible that the respondents made false statements in the sense of a social desirability bias (64). This should be questioned in particular, as the relevance and performance level of both target groups were of close-range and the respondents almost exclusively stated a good level of competency. In our analysis, we identified significant differences in training needs between subgroups. However, we conducted a cross-sectional study that cannot prove causalities and directions of effect. Furthermore, a different definition of subgroups, e.g., with regard to age groups, might have led to different results. It is noteworthy that the level of education of the respondents was rather high in both samples. However, no statements can be made about the representativeness of the samples and thus the transferability of results, as there is no reliable information on the level of education of telerehabilitation aftercare patients and therapists in Germany. At least in comparison to other studies from Germany on telerehabilitation aftercare, the educational status of patients in our study is higher (65, 66). At the same time, the voluntary participation may have resulted in a self-selection bias, which could have led to particularly technology affine people taking part in the survey and thus causing training needs to be underestimated. The aim was to reach the complete target population of therapists and patients in Germany who are participating in telerehabilitation aftercare. However, there are potential biases due to recruitment, as the referral process in the contacted facilities was not transparent and the actual coverage of the target population therefore remains unclear. Also, the small sample size is a limitation of the study. We have shown that different types of telerehabilitation can be associated with different training needs. It is important to note here that the individual programs have different technical features and content that might need different training. These differences were not captured in our study because we analyzed groups of program types. Lastly, it should be noted that the identified training needs relate to the existing telerehabilitation programs for rehabilitation aftercare in Germany and that transferability to other care settings with other digital tools in other countries may be limited.

### 4.4 Conclusion

Both target groups rated their own performance of the telerehabilitation competencies mainly as rather good. Nevertheless, around two-thirds of the patient competencies and around one-third of the therapist competencies showed a small need for training. The findings can be used to optimize existing training options or develop new training programs to better prepare patients and therapists for telerehabilitation. These should address the greatest training needs and should be tailored to the individual contexts and needs of the user groups—e.g. regarding the type of program used and the users’ affinity for technology.

## Data Availability

The original contributions presented in the study are included in the article/Supplementary material, further inquiries can be directed to the corresponding author.
