# Supplementary material for: Training needs in telerehabilitation: results of a cross-sectional online survey with therapists and patients
Source: Front Public Health. 2025 Dec 11;13:1688055. doi: 10.3389/fpubh.2025.1688055 (PMC12738953; doi:10.3389/fpubh.2025.1688055)
Supplement: Supplementary file 2 [file Supplementary_file_2.pdf]

S2 appendix. Online surveys for patients and therapists

Online survey for telerehabilitation aftercare patients

Welcome to the online survey as part of the "TelReKo" research project

Dear participants,
Thank you for taking the time to participate in our survey. You are making a valuable contribution to our research project.

What is the survey about?
With our survey, we would like to ascertain the skills required for the successful use of tele-rehabilitation aftercare programs. We would also like to find out how you as a patient have been prepared and trained to use tele-rehab aftercare. Finally, we would like to compare the skills you have indicated with the existing training programs in order to identify training needs and requirements. These results can be used to adapt future training offers even better to the needs and requirements of users and thus ensure the successful use of tele-rehabilitation aftercare.
The data collection is part of the "TelReKo" research project (training needs and requirements of therapists and rehabilitants for competent use of online-based rehabilitation aftercare services). The project is being carried out by the University of Siegen under the direction of Prof. Dr. C. Dockweiler and is funded by the Gesellschaft für Rehabilitationswissenschaften NRW e.V. (GfR).

Who can take part?
You can take part in the survey if you have used a tele-rehab aftercare program (e.g. enlivio, Caspar, DE-RENA or others) as a patient within the last two years. Please also complete the questionnaire if you no longer use the program. These experiences are also of interest to us.
The survey takes about 15 minutes to complete.
If you would like to go back to a previous question, please use the white "Back" button at the bottom of each page and not the back function of your browser. If you interrupt the survey and wish to continue at another time, you must temporarily save the survey or your previous answers. To do this, click on "Continue later" at the top right of the survey window and follow the instructions displayed.

Data protection
All data will be processed, stored and analyzed in compliance with the applicable data protection regulations. In order to participate in the survey, you must agree to these regulations. To do so, please check the appropriate box at the bottom of this page.
Participation in the survey is voluntary. You can end it at any time without giving reasons and without incurring any disadvantages. Your participation is anonymous, i.e. the data collected does not allow any conclusions to be drawn about your person. The data will not be passed on to other persons or institutions (such as clinics or rehabilitation centers). After completion of the study, the research data collected will be published in anonymized form, e.g. in the form of scientific articles in specialist journals.
If you have any questions about participation, voluntariness and data use, please contact Stephan Krayter (stephan.krayter@uni-siegen.de) or Lea Stark (lea.stark@uni-siegen.de).

Part 1 - Information on the tele-rehab aftercare program

Are you currently using a tele-rehab aftercare program? *

Please select only one of the following answers:

- ◯ Yes
- ◯ No

How long ago was it last used?

*

Only answer this question if the following conditions are met:
Answer was 'No' to question '[G03Q05]' (Do you currently use a tele-rehab aftercare program?)
Please select only one of the following answers:

- ◯ Less than one month
- ◯ Between one and less than three months
- ◯ Between three and less than six months
- ◯ Six months or longer

What type of tele-rehabilitation aftercare do you use or have you used?

*

Please select only one of the following answers:

- ◯ Independent use of the app/program by patients
- ◯ Video conference guided by therapists

Do you use wearables in this program (e.g. pedometers, blood pressure monitors, etc.) or have you used any?

*

Please select only one of the following answers:

- ◯ Yes
- ◯ No

Which indication group does the program refer to?

*

Please select all applicable answers:

- ☐ Psychosomatics
- ☐ Orthopaedics
- ☐ Cardiology
- ☐ Neurology
- ☐ Oncology
- ☐ Pulmonology
- ☐ Other

Have you completed the program to the intended end?

*

Only answer this question if the following conditions are met:
((G03Q05.NAOK (/index.php/questionAdministration/view/surveyid/715987/gid/17047/qid/206124) == "AO02"))
Please select only one of the following answers:

- ◯ Yes
- ◯ No

Were difficulties in use the main reason for the premature termination?

*

Only answer this question if the following conditions are met:
Answer was 'No' to question '[G01Q09]' (Did you complete the program to the intended end?)
Please select only one of the following answers:

- ◯ Yes
- ◯ No

How long have you been using the program or have you used it in total? *

Please select only one of the following answers:

- ◯ Less than one month
- ◯ Between one and less than three months
- ◯ Between three and less than six months
- ◯ Six months or longer

How often do you use or have you used the program on average?

*

Please select only one of the following answers:

- ◯ Daily
- ◯ Several times a week
- ◯ Once a week
- ◯ Several times a month
- ◯ Once a month or less

Part 2 - Steps in tele-rehab aftercare

What steps did you go through in preparation for tele-rehab aftercare?

*

Please select all applicable answers:

- ☐ Independent information about the program
- ☐ Participation in a discussion/event with information about the program
- ☐ Participation in a practical introduction to the program
- ☐ Technical setup of the program
- ☐ Individual adaptation of the therapy to own needs (if necessary with therapist)
- ☐ Other:

What steps are you going through or have you gone through during the program?

*

Please select all applicable answers:

- ☐ Following the therapist's instructions
- ☐ Individual adaptation of therapy to own needs (e.g. selecting content, difficulty)
- ☐ Dealing with health problems (e.g. certain symptoms or emergencies)
- ☐ Solving technical problems
- ☐ Self-monitoring (e.g. therapy progress, health parameters)
- ☐ Motivating yourself
- ☐ Reminding themselves (e.g. of therapy implementation or appointments)
- ☐ Own documentation of therapy sessions
- ☐ Other:

Do you see any of these steps as being the responsibility of therapists or other people?

*

Please select only one of the following answers:

◯ No

◯ Yes:

Part 3 - Skills required in tele-rehabilitation aftercare

The following section deals with the skills you need as a patient to use the tele-rehab aftercare program. The areas of knowledge, skills, attitudes and previous experience are discussed below. For each competence, you are asked to provide two answers on a scale of 1-7. Please answer how relevant you consider the competence to be for patients (1 = not at all important; 7 = very important) and how pronounced you consider this competence to be for yourself (1 = not at all pronounced; 7 = very pronounced).

Knowledge *

Please select the appropriate answer for each item:

	How relevant is this knowledge?								How pronounced is this knowledge in my case?						
	1	2	3	4	5	6	7		1	2	3	4	5	6	7
**Knowledge about tele-rehab aftercare (e.g. content & procedures)**	◯	◯	◯	◯	◯	◯	◯		◯	◯	◯	◯	◯	◯	◯
**Legal knowledge (e.g. data protection)**	◯	◯	◯	◯	◯	◯	◯		◯	◯	◯	◯	◯	◯	◯
**Technical knowledge (e.g. installation & troubleshooting)**	◯	◯	◯	◯	◯	◯	◯		◯	◯	◯	◯	◯	◯	◯
**Medical knowledge (e.g. clinical picture & procedure for medical emergencies)**	◯	◯	◯	◯	◯	◯	◯		◯	◯	◯	◯	◯	◯	◯

Skills *

Please select the appropriate answer for each item:

	How relevant is this knowledge?								How pronounced is this knowledge in my case?						
	1	2	3	4	5	6	7		1	2	3	4	5	6	7
**Technical skills (e.g. setting-up using, adapting technology)**	◯	◯	◯	◯	◯	◯	◯		◯	◯	◯	◯	◯	◯	◯
**Adaptability (anticipating and perceiving changes, adapting flexibly to them)**	◯	◯	◯	◯	◯	◯	◯		◯	◯	◯	◯	◯	◯	◯
**Reflection skills (questioning oneself and others, reflecting critically)**	◯	◯	◯	◯	◯	◯	◯		◯	◯	◯	◯	◯	◯	◯
**Analytical skills (understanding and solving problems)**	◯	◯	◯	◯	◯	◯	◯		◯	◯	◯	◯	◯	◯	◯
**Empathy (being able to identify with and feel for others)**	◯	◯	◯	◯	◯	◯	◯		◯	◯	◯	◯	◯	◯	◯
**Teamwork skills (working constructively with others)**	◯	◯	◯	◯	◯	◯	◯		◯	◯	◯	◯	◯	◯	◯
**Communication skills (being able and willing to communicate with others)**	◯	◯	◯	◯	◯	◯	◯		◯	◯	◯	◯	◯	◯	◯
**Motivational skills (motivating yourself and others)**	◯	◯	◯	◯	◯	◯	◯		◯	◯	◯	◯	◯	◯	◯
**Self-management skills (e.g. ability to work independently)**	◯	◯	◯	◯	◯	◯	◯		◯	◯	◯	◯	◯	◯	◯
**Patience**	◯	◯	◯	◯	◯	◯	◯		◯	◯	◯	◯	◯	◯	◯
**Self-awareness (being aware of yourself and your own thoughts, feelings and actions)**	◯	◯	◯	◯	◯	◯	◯		◯	◯	◯	◯	◯	◯	◯
**Reading and writing skills**	◯	◯	◯	◯	◯	◯	◯		◯	◯	◯	◯	◯	◯	◯

Attitudes *

Please select the appropriate answer for each item:

	How relevant is this knowledge?								How pronounced is this knowledge in my case?						
	1	2	3	4	5	6	7		1	2	3	4	5	6	7
**Affinity for technology (e.g. enthusiasm for technology)**	◯	◯	◯	◯	◯	◯	◯		◯	◯	◯	◯	◯	◯	◯
**Technology acceptance**	◯	◯	◯	◯	◯	◯	◯		◯	◯	◯	◯	◯	◯	◯
**Willingness to learn**	◯	◯	◯	◯	◯	◯	◯		◯	◯	◯	◯	◯	◯	◯
**Openness towards new things**	◯	◯	◯	◯	◯	◯	◯		◯	◯	◯	◯	◯	◯	◯
**Frustration tolerance**	◯	◯	◯	◯	◯	◯	◯		◯	◯	◯	◯	◯	◯	◯
**Self-efficacy expectation (conviction of being able to carry out actions successfully)**	◯	◯	◯	◯	◯	◯	◯		◯	◯	◯	◯	◯	◯	◯
**Self-interest in the tele-rehab aftercare program**	◯	◯	◯	◯	◯	◯	◯		◯	◯	◯	◯	◯	◯	◯

Experience *

Please select the appropriate answer for each item:

	How relevant is this knowledge?								How pronounced is this knowledge in my case?						
	1	2	3	4	5	6	7		1	2	3	4	5	6	7
**Experience in analog therapy**	◯	◯	◯	◯	◯	◯	◯		◯	◯	◯	◯	◯	◯	◯
**Experience in the use of digital health-related applications (e.g. health apps)**	◯	◯	◯	◯	◯	◯	◯		◯	◯	◯	◯	◯	◯	◯
**Experience in the use of digital devices (e.g. computer, tablet, smartwatch)**	◯	◯	◯	◯	◯	◯	◯		◯	◯	◯	◯	◯	◯	◯

In your opinion, do the following patient characteristics influence the successful use of tele-rehab aftercare?

*

Please select the appropriate answer for each item:

	Yes	No	I cannot/would not like to make a comment on this
**Age**	◯	◯	◯
**Gender**	◯	◯	◯
**Level of education**	◯	◯	◯
**Language abilities**	◯	◯	◯
**Socio-economic status**	◯	◯	◯
**Place of residence (urban vs. rural)**	◯	◯	◯
**Severity of your own illness**	◯	◯	◯

Are there other competencies (knowledge, skills, attitudes, personality traits) that you consider relevant?

*

Please select only one of the following answers:

◯ No

◯ Yes

Part 4 - Preparing patients for the use of tele-rehab aftercare

What options were/are available to you for preparing and assisting with the tele-rehab aftercare program?

*

Please select the appropriate answer for each item:

	Was offered, I made use of it	Was offered, I did not use it	Was not offered, nor was it necessary	Was not offered, I would have liked it
**Individual consultation with therapist or doctor**	◯	◯	◯	◯
**On-site presentation**	◯	◯	◯	◯
**On-site workshop with the opportunity to try out the program**	◯	◯	◯	◯
**Online presentation**	◯	◯	◯	◯
**Online webinar (interactive)**	◯	◯	◯	◯
**Information videos**	◯	◯	◯	◯
**Written information material**	◯	◯	◯	◯
**One-to-one consultation options (e.g. by phone, email/chat)**	◯	◯	◯	◯

Part 5 - Training needs

To what extent have the following areas of knowledge been covered in previous training offers (1 = not at all; 7 = completely)?

*

Please select the appropriate answer for each item:

	1	2	3	4	5	6	7
**Knowledge about tele-rehab aftercare (e.g. content & procedures)**	◯	◯	◯	◯	◯	◯	◯
**Legal knowledge (e.g. data protection)**	◯	◯	◯	◯	◯	◯	◯
**Technical knowledge (e.g. installation & troubleshooting)**	◯	◯	◯	◯	◯	◯	◯
**Medical knowledge (e.g. clinical picture & procedure for medical emergencies)**	◯	◯	◯	◯	◯	◯	◯

To what extent have the following skills been covered in previous training offers (1 = not at all; 7 = completely)?
*

Please select the appropriate answer for each item:

	1	2	3	4	5	6	7
**Technical skills (e.g. setting-up using, adapting technology)**	◯	◯	◯	◯	◯	◯	◯
**Adaptability (anticipating and perceiving changes, adapting flexibly to them)**	◯	◯	◯	◯	◯	◯	◯
**Reflection skills (questioning oneself and others, reflecting critically)**	◯	◯	◯	◯	◯	◯	◯
**Analytical skills (understanding and solving problems)**	◯	◯	◯	◯	◯	◯	◯
**Empathy (being able to identify with and feel for others)**	◯	◯	◯	◯	◯	◯	◯
**Teamwork skills (working constructively with others)**	◯	◯	◯	◯	◯	◯	◯
**Communication skills (being able and willing to communicate with others)**	◯	◯	◯	◯	◯	◯	◯
**Motivational skills (motivating yourself and others)**	◯	◯	◯	◯	◯	◯	◯
**Self-management skills (e.g. ability to work independently)**	◯	◯	◯	◯	◯	◯	◯
**Patience**	◯	◯	◯	◯	◯	◯	◯
**Self-awareness (being aware of yourself and your own thoughts, feelings and actions)**	◯	◯	◯	◯	◯	◯	◯
**Reading and writing skills**	◯	◯	◯	◯	◯	◯	◯

To what extent have the following attitudes been covered in previous training offers (1 = not at all; 7 = completely)?
*

Please select the appropriate answer for each item:

	1	2	3	4	5	6	7
**Affinity for technology (e.g. enthusiasm for technology)**	◯	◯	◯	◯	◯	◯	◯
**Technology acceptance**	◯	◯	◯	◯	◯	◯	◯
**Willingness to learn**	◯	◯	◯	◯	◯	◯	◯
**Openness towards new things**	◯	◯	◯	◯	◯	◯	◯
**Frustration tolerance**	◯	◯	◯	◯	◯	◯	◯
**Self-efficacy expectation (conviction of being able to carry out actions successfully)**	◯	◯	◯	◯	◯	◯	◯
**Self-interest in the tele-rehab aftercare program**	◯	◯	◯	◯	◯	◯	◯

Part 6 - Affinity for technology

In the following questionnaire, we will ask you about your interaction with technical systems. The term “technical systems” refers to apps and other software applications, as well as entire digital devices (e.g., mobile phone, computer, TV, car navigation).

Please indicate your level of consent

*

Please select the appropriate answer for each item:

	completely disagree	largely disagree	slightly disagree	slightly agree	largely agree	completely agree
**I like to occupy myself in greater detail with technical systems.**	◯	◯	◯	◯	◯	◯
**I like testing the functions of new technical systems.**	◯	◯	◯	◯	◯	◯
**I predominantly deal with technical systems because I have to.**	◯	◯	◯	◯	◯	◯
**When I have a new technical system in front of me, I try it out intensively.**	◯	◯	◯	◯	◯	◯
**I enjoy spending time becoming acquainted with a new technical system.**	◯	◯	◯	◯	◯	◯
**It is enough for me that a technical system works; I don't care how or why.**	◯	◯	◯	◯	◯	◯
**I try to understand how a technical system exactly works.**	◯	◯	◯	◯	◯	◯
**It is enough for me to know the basic functions of a technical system.**	◯	◯	◯	◯	◯	◯
**I try to make full use of the capabilities of a technical system.**	◯	◯	◯	◯	◯	◯

Part 7 - Socio-demographic data

In the last section, we would like to ask you for some personal details. This information will of course also be evaluated anonymously. By answering the questions, you will help us to make training courses in this area more target group-specific in the future (e.g. age group-specific).

How old are you? *

Please select only one of the following answers:

- ◯ <20
- ◯ 20-29
- ◯ 30-39
- ◯ 40-49
- ◯ 50-59
- ◯ 60-69
- ◯ 70-79
- ◯ 80-89
- ◯ ≥90
- ◯ I do not wish to give any detailsn

Which gender do you identify with? *

Please select only one of the following answers:

- ◯ Male
- ◯ Female
- ◯ Divers
- ◯ I do not wish to give any details

What is your highest educational or professional qualification?

*

Please select only one of the following answers:

- ◯ No educational qualifications
- ◯ Hauptschulabschluss (lower secondary school qualification)
- ◯ “Mittlere Reife” (secondary school certicicate)
- ◯ “Fachhochschulreife” (specialised A-levels)
- ◯ “Allgemeine Hochschulreife” (A-levels)
- ◯ Vocational training
- ◯ Bachelor's degree / equivalent educational program
- ◯ Master's degree / equivalent educational program
- ◯ Promotion
- ◯ I do not wish to give any details

Do you have people around you who can support you in using the tele-rehab aftercare program?

*

Please select only one of the following answers:

- ☐ Yes
- ☐ No

End of survey

You have reached the end of the survey. Click on "Submit" to end the survey. You will then no longer have the opportunity to revise your answers. Click on "Back" to return to the previous survey page if you wish to revise or add to your answers.

Thank you very much for taking the time to complete the survey!

You are supporting our project greatly and contributing to the further development of research and practice in tele-rehabilitation aftercare in Germany.

For the further course of our study, we are looking for therapists, rehabilitants and technology providers who would like to discuss the topic of "Training in tele-rehabilitation aftercare" with us in a group format.

If you are interested, please contact: Stephan Krayter (stephan.krayter@uni-siegen.de) or Lea Stark (lea.stark@uni-siegen.de). Your contact details will not be stored in connection with your participation in this survey.

Online survey for telerehabilitation aftercare therapists

Welcome to the online survey as part of the "TelReKo" research project

Dear participants,
Thank you for taking the time to participate in our survey. You are making a valuable contribution to our research project.

What is the survey about?
With our survey, we would like to ascertain the skills required for the successful use of tele-rehabilitation aftercare programs. We would also like to find out how you as a therapist have been prepared and trained to practice tele-rehab aftercare. Finally, we would like to compare the skills you have indicated you need with existing training programs in order to identify training needs and requirements.
These results can be used to adapt future training offers even better to the needs and requirements of users and thus ensure the successful use of tele-rehabilitation aftercare.
The data collection is part of the "TelReKo" research project (training needs and requirements of therapists and rehabilitants for competent use of online-based rehabilitation aftercare services). The project is being carried out by the University of Siegen under the direction of Prof. Dr. C. Dockweiler and is funded by the Gesellschaft für Rehabilitationswissenschaften NRW e.V. (GfR).

Who can take part?
You can take part in the survey if you have used a tele-rehab aftercare program (e.g. enlivio, Caspar, DE-RENA or others) as a therapist within the last two years. Please also complete the questionnaire if you no longer use the program. These experiences are also of interest to us.
The survey takes about 15 minutes to complete.
If you would like to go back to a previous question, please use the white "Back" button at the bottom of each page and not the back function of your browser. If you interrupt the survey and wish to continue at another time, you must temporarily save the survey or your previous answers. To do this, click on "Continue later" at the top right of the survey window and follow the instructions displayed.

Data protection
All data will be processed, stored and analyzed in compliance with the applicable data protection regulations. In order to participate in the survey, you must agree to these regulations. To do so, please check the appropriate box at the bottom of this page.
Participation in the survey is voluntary. You can end it at any time without giving reasons and without incurring any disadvantages.
Your participation is anonymous, i.e. the data collected does not allow any conclusions to be drawn about your person. The data will not be passed on to other persons or institutions (such as clinics or rehabilitation centers). After completion of the study, the research data collected will be published in anonymized form, e.g. in the form of scientific articles in specialist journals.
If you have any questions about participation, voluntariness and data use, please contact Stephan Krayter (stephan.krayter@uni-siegen.de) or Lea Stark (lea.stark@uni-siegen.de).

Part 1 - Information on the tele-rehab aftercare program and your role as a therapist

Are you currently using a tele-rehab aftercare program?
*

Please select only one of the following answers:

◯ Yes
◯ No

How long ago was it last used? *

Please select only one of the following answers:

◯ Less than one month
◯ Between one and less than three months
◯ Between three and less than six months
◯ Six months or longer

What type of tele-rehabilitation aftercare do you use or have you used?

*

Please select only one of the following answers:

◯ Independent use of the app/program by patients
◯ Video conference guided by therapists

Are or were wearables (e.g. pedometers, blood pressure monitors, etc.) used by patients in this program?

*

Please select only one of the following answers:

◯ Yes
◯ No

Which indication group does the program refer to?

*

Please select all applicable answers:

☐ Psychosomatics
☐ Orthopaedics
☐ Cardiology
☐ Neurology
☐ Oncology
☐ Pulmonology
☐ Other

What type of therapy do you practise?

*

Please select all applicable answers:

☐ Psychotherapy
☐ Physiotherapy
☐ Sports/movement therapy
☐ Speech therapy
☐ Occupational therapy
☐ Other therapies

How long have you been using the program or have you used it in total?

*

Please select only one of the following answers:

◯ Less than one month
◯ Between one and less than three months
◯ Between three and less than six months
◯ Six months or longer

How often do you use or have you used the program on average?

*

Please select only one of the following answers:

- ◯ Daily
- ◯ Several times a week
- ◯ Once a week
- ◯ Several times a month
- ◯ Once a month or less

In what form do you work as a therapist?

*

Please select only one of the following answers:

- ◯ Exclusively tele-therapist
- ◯ Tele-therapist and analog therapist

How long have you been working as a therapist?

*

Please select only one of the following answers:

- ◯ Less than one year
- ◯ Between one and less than three years
- ◯ Between three and less than five years
- ◯ Five years or more

What is the correct description for the institution in which you work?

*

Please select only one of the following answers:

- ◯ Tele-Therapy Clinic
- ◯ Inpatient rehabilitation facility
- ◯ Outpatient practice

Part 2 - Tasks of therapists in tele-rehabilitation aftercare

What work tasks will you take on or have you taken on before the program starts?

*

Please select all applicable answers:

- ☐ Informing patients about the program
- ☐ Practical instruction of patients in the program
- ☐ Technical setup of the program
- ☐ Supporting patients with the technical set-up
- ☐ Individual adaptation of the therapy to the patient's needs
- ☐ Other: ____

What work tasks will you take on or have you taken on during the program?

*

Please select all applicable answers:

- ☐ Guidance for patients
- ☐ Adapting the therapy to the patient's needs
- ☐ Support with patients' health problems
- ☐ Solving technical problems
- ☐ Monitoring patients (e.g. therapy progress, health parameters)
- ☐ Giving feedback or motivation
- ☐ Reminding patients (e.g. of therapy implementation or appointments)
- ☐ Documentation of therapy sessions
- ☐ Other: [ ]

Do you see any of these tasks as being the responsibility of patients or other people?

*

Please select all applicable answers:

- ◯ No
- ◯ Yes: [ ]

Part 3 - Skills required in tele-rehabilitation aftercare

The following section deals with the skills you need as a therapist to use the tele-rehab aftercare program. The areas of knowledge, skills, attitudes and previous experience are discussed below. For each competency, you are asked to provide two answers on a scale of 1-7. Please answer how relevant you consider the competence to be for therapists (1 = not at all important; 7 = very important) and how pronounced you consider this competence to be for yourself (1 = not at all pronounced; 7 = very pronounced).

Knowledge *

Please select the appropriate answer for each item:

	How relevant is this knowledge?								How pronounced is this knowledge in my case?						
	1	2	3	4	5	6	7		1	2	3	4	5	6	7
**Knowledge about tele-rehab aftercare (e.g. content & procedures)**	◯	◯	◯	◯	◯	◯	◯		◯	◯	◯	◯	◯	◯	◯
**Knowledge about implementation (e.g. policies & processes)**	◯	◯	◯	◯	◯	◯	◯		◯	◯	◯	◯	◯	◯	◯
**Legal knowledge (e.g. data protection)**	◯	◯	◯	◯	◯	◯	◯		◯	◯	◯	◯	◯	◯	◯
**Technical knowledge (e.g. installation & troubleshooting)**	◯	◯	◯	◯	◯	◯	◯		◯	◯	◯	◯	◯	◯	◯
**Medical knowledge (e.g. clinical picture & procedure for medical emergencies)**	◯	◯	◯	◯	◯	◯	◯		◯	◯	◯	◯	◯	◯	◯
**Process-related knowledge (e.g. quality assurance and management)**	◯	◯	◯	◯	◯	◯	◯		◯	◯	◯	◯	◯	◯	◯

Skills *

Please select the appropriate answer for each item:

	How relevant is this knowledge?								How pronounced is this knowledge in my case?						
	1	2	3	4	5	6	7		1	2	3	4	5	6	7
**Technical skills (e.g. setting-up using, adapting technology)**	◯	◯	◯	◯	◯	◯	◯		◯	◯	◯	◯	◯	◯	◯
**Therapeutic-professional skills (carrying out the therapy professionally)**	◯	◯	◯	◯	◯	◯	◯		◯	◯	◯	◯	◯	◯	◯
**Adaptability (anticipating and perceiving changes, adapting flexibly to them)**	◯	◯	◯	◯	◯	◯	◯		◯	◯	◯	◯	◯	◯	◯
**Reflection skills (questioning oneself and others, reflecting critically)**	◯	◯	◯	◯	◯	◯	◯		◯	◯	◯	◯	◯	◯	◯
**Analytical skills (understanding and solving problems)**	◯	◯	◯	◯	◯	◯	◯		◯	◯	◯	◯	◯	◯	◯
**Empathy (being able to identify with and feel for others)**	◯	◯	◯	◯	◯	◯	◯		◯	◯	◯	◯	◯	◯	◯
**Teamwork skills (working constructively with others)**	◯	◯	◯	◯	◯	◯	◯		◯	◯	◯	◯	◯	◯	◯
**Communication skills (being able and willing to communicate with others)**	◯	◯	◯	◯	◯	◯	◯		◯	◯	◯	◯	◯	◯	◯
**Motivational skills (motivating yourself and others)**	◯	◯	◯	◯	◯	◯	◯		◯	◯	◯	◯	◯	◯	◯
**Self-management skills (e.g. ability to work independently)**	◯	◯	◯	◯	◯	◯	◯		◯	◯	◯	◯	◯	◯	◯
**Patience**	◯	◯	◯	◯	◯	◯	◯		◯	◯	◯	◯	◯	◯	◯
**Self-awareness (being aware of yourself and your own thoughts, feelings and actions)**	◯	◯	◯	◯	◯	◯	◯		◯	◯	◯	◯	◯	◯	◯

Attitudes

*

Please select the appropriate answer for each item:

	How relevant is this knowledge?								How pronounced is this knowledge in my case?						
	1	2	3	4	5	6	7		1	2	3	4	5	6	7
**Affinity for technology (e.g. enthusiasm for technology)**	◯	◯	◯	◯	◯	◯	◯		◯	◯	◯	◯	◯	◯	◯
**Technology acceptance**	◯	◯	◯	◯	◯	◯	◯		◯	◯	◯	◯	◯	◯	◯
**Willingness to learn**	◯	◯	◯	◯	◯	◯	◯		◯	◯	◯	◯	◯	◯	◯
**Openness towards new things**	◯	◯	◯	◯	◯	◯	◯		◯	◯	◯	◯	◯	◯	◯
**Frustration tolerance**	◯	◯	◯	◯	◯	◯	◯		◯	◯	◯	◯	◯	◯	◯
**Self-efficacy expectation (conviction of being able to carry out actions successfully)**	◯	◯	◯	◯	◯	◯	◯		◯	◯	◯	◯	◯	◯	◯
**Self-interest in the tele-rehab aftercare program**	◯	◯	◯	◯	◯	◯	◯		◯	◯	◯	◯	◯	◯	◯

Experience *

Please select the appropriate answer for each item:

	How relevant is this knowledge?								How pronounced is this knowledge in my case?						
	1	2	3	4	5	6	7		1	2	3	4	5	6	7
**Work experience in an analog therapy**	○	○	○	○	○	○	○		○	○	○	○	○	○	○
**Experience in the use of digital applications at work (e.g. therapy software, resource planning)**	○	○	○	○	○	○	○		○	○	○	○	○	○	○
**Experience in the use of digital devices (e.g. computer, tablet, smartwatch)**	○	○	○	○	○	○	○		○	○	○	○	○	○	○

In your opinion, do the following therapist characteristics influence the successful use of tele-rehab aftercare?

*

Please select the appropriate answer for each item:

	Yes	No	I cannot/would not like to make a comment on this
**Age**	○	○	○
**Gender**	○	○	○
**Level of education**	○	○	○
**Language abilities**	○	○	○
**Socio-economic status**	○	○	○
**Form of therapy practiced**	○	○	○

Are there other competencies (knowledge, skills, attitudes, personality traits) that you consider relevant?

*

Please select only one of the following answers:

○ No

○ Yes:

Part 4 - Preparing therapists for the use of tele-rehab aftercare

What options were/are available to you for preparing and assisting with the tele-rehab aftercare program?

*

Please select the appropriate answer for each item:

	Was offered, I made use of it	Was offered, I did not use it	Was not offered, nor was it necessary	Was not offered, I would have liked it
**Module/lecture in vocational training or university**	◯	◯	◯	◯
**Certified training/ advanced training in the field of telemedicine/ telerehabilitation**	◯	◯	◯	◯
**On-site presentation**	◯	◯	◯	◯
**On-site workshop with the opportunity to try out the program**	◯	◯	◯	◯
**Online presentation**	◯	◯	◯	◯
**Online webinar (interactive)**	◯	◯	◯	◯
**Information videos**	◯	◯	◯	◯
**Written information material**	◯	◯	◯	◯
**One-to-one consultation options (e.g. by phone, email/chat)**	◯	◯	◯	◯

Part 5 - Training Needs

To what extent have the following areas of knowledge been covered in previous training offers (1 = not at all; 7 = completely)?

*

Please select the appropriate answer for each item:

	1	2	3	4	5	6	7
**Knowledge about tele-rehab aftercare (e.g. content & procedures)**	◯	◯	◯	◯	◯	◯	◯
**Knowledge about implementation (e.g. policies & processes)**	◯	◯	◯	◯	◯	◯	◯
**Legal knowledge (e.g. data protection)**	◯	◯	◯	◯	◯	◯	◯
**Technical knowledge (e.g. installation & troubleshooting)**	◯	◯	◯	◯	◯	◯	◯
**Medical knowledge (e.g. clinical picture & procedure for medical emergencies)**	◯	◯	◯	◯	◯	◯	◯
**Process-related knowledge (e.g. quality assurance and management)**	◯	◯	◯	◯	◯	◯	◯

To what extent have the following skills been covered in previous training offers (1 = not at all; 7 = completely)?

*

Please select the appropriate answer for each item:

	1	2	3	4	5	6	7
**Technical skills (e.g. setting-up using, adapting technology)**	◯	◯	◯	◯	◯	◯	◯
**Therapeutic-professional skills (carrying out the therapy professionally)**	◯	◯	◯	◯	◯	◯	◯
**Adaptability (anticipating and perceiving changes, adapting flexibly to them)**	◯	◯	◯	◯	◯	◯	◯
**Reflection skills (questioning oneself and others, reflecting critically)**	◯	◯	◯	◯	◯	◯	◯
**Analytical skills (understanding and solving problems)**	◯	◯	◯	◯	◯	◯	◯
**Empathy (being able to identify with and feel for others)**	◯	◯	◯	◯	◯	◯	◯
**Teamwork skills (working constructively with others)**	◯	◯	◯	◯	◯	◯	◯
**Communication skills (being able and willing to communicate with others)**	◯	◯	◯	◯	◯	◯	◯
**Motivational skills (motivating yourself and others)**	◯	◯	◯	◯	◯	◯	◯
**Self-management skills (e.g. ability to work independently)**	◯	◯	◯	◯	◯	◯	◯
**Patience**	◯	◯	◯	◯	◯	◯	◯
**Self-awareness (being aware of yourself and your own thoughts, feelings and actions)**	◯	◯	◯	◯	◯	◯	◯

To what extent have the following attitudes been covered in previous training offers (1 = not at all; 7 = completely)?

*

Please select the appropriate answer for each item:

	1	2	3	4	5	6	7
**Affinity for technology (e.g. enthusiasm for technology)**	◯	◯	◯	◯	◯	◯	◯
**Technology acceptance**	◯	◯	◯	◯	◯	◯	◯
**Willingness to learn**	◯	◯	◯	◯	◯	◯	◯
**Openness towards new things**	◯	◯	◯	◯	◯	◯	◯
**Frustration**	◯	◯	◯	◯	◯	◯	◯
**Self-efficacy expectation (conviction of being able to carry out actions successfully)**	◯	◯	◯	◯	◯	◯	◯
**Self-interest in the tele-rehab aftercare program**	◯	◯	◯	◯	◯	◯	◯

Part 6 - Affinity for technology

In the following questionnaire, we will ask you about your interaction with technical systems. The term “technical systems” refers to apps and other software applications, as well as entire digital devices (e.g., mobile phone, computer, TV, car navigation).

Please indicate your level of consent *

Please select the appropriate answer for each item:

	completely disagree	largely disagree	slightly disagree	slightly agree	largely agree	completely agree
**I like to occupy myself in greater detail with technical systems.**	◯	◯	◯	◯	◯	◯
**I like testing the functions of new technical systems.**	◯	◯	◯	◯	◯	◯
**I predominantly deal with technical systems because I have to.**	◯	◯	◯	◯	◯	◯
**When I have a new technical system in front of me, I try it out intensively.**	◯	◯	◯	◯	◯	◯
**I enjoy spending time becoming acquainted with a new technical system.**	◯	◯	◯	◯	◯	◯
**It is enough for me that a technical system works; I don't care how or why.**	◯	◯	◯	◯	◯	◯
**I try to understand how a technical system exactly works.**	◯	◯	◯	◯	◯	◯
**It is enough for me to know the basic functions of a technical system.**	◯	◯	◯	◯	◯	◯
**Il try to make full use of the capabilities of a technical system.**	◯	◯	◯	◯	◯	◯

Part 7 - Socio-demographic data

In the last section, we would like to ask you for some personal details. This information will of course also be evaluated anonymously. By answering the questions, you will help us to make training courses in this area more target group-specific in the future (e.g. age group-specific).

How old are you?

*

Please select only one of the following answers:

- ◯ <20
- ◯ 20-29
- ◯ 30-39
- ◯ 40-49
- ◯ 50-59
- ◯ 60-69
- ◯ 70-79
- ◯ 80-89
- ◯ ≥90
- ◯ I do not wish to give any details

Which gender do you identify with?

*

Please select only one of the following answers:

- ◯ Male
- ◯ Female
- ◯ Divers
- ◯ I do not wish to give any details

What is your highest educational or professional qualification?

*

Please select only one of the following answers:

- ◯ No educational qualifications
- ◯ Hauptschulabschluss (lower secondary school qualification)
- ◯ “Mittlere Reife” (secondary school certicicate)
- ◯ “Fachhochschulreife” (specialised A-levels)
- ◯ “Allgemeine Hochschulreife” (A-levels)
- ◯ Vocational training
- ◯ Bachelor's degree / equivalent educational program
- ◯ Master's degree / equivalent educational program
- ◯ Promotion
- ◯ I do not wish to give any details

End of survey

You have reached the end of the survey. Click on "Submit" to end the survey. You will then no longer have the opportunity to revise your answers. Click on "Back" to return to the previous survey page if you wish to revise or add to your answers.

Thank you very much for taking the time to complete the survey!

You are supporting our project greatly and contributing to the further development of research and practice in tele-rehabilitation aftercare in Germany.

For the further course of our study, we are looking for therapists, rehabilitants and technology providers who would like to discuss the topic of "Training in tele-rehabilitation aftercare" with us in a group format.

If you are interested, please contact: Stephan Krayter (stephan.krayter@uni-siegen.de) or Lea Stark (lea.stark@uni-siegen.de). Your contact details will not be stored in connection with your participation in this survey.
