# Supplementary material for: Training needs in telerehabilitation: results of a cross-sectional online survey with therapists and patients
Source: Front Public Health. 2025 Dec 11;13:1688055. doi: 10.3389/fpubh.2025.1688055 (PMC12738953; doi:10.3389/fpubh.2025.1688055)
Supplement: Supplementary file 3 [file Supplementary_file_3.pdf]

S3 appendix. Group variables, Shapiro-Wilk- and Levene-Tests

Table S3a. Group variables and group size according to target group ($n_{patients}$=262, $n_{therapists}$=73, adjusted data set)

	Patients (N=262)				Therapists (N=73)			
Group Variable	Group 1	Group 2	Size Group 1	Size Group 2	Group 1	Group 2	Size Group 1	Size Group 2
Age	<50	≥50	70	192	<40	≥40	35	38
Gender	Men	Women	62	200	Men	Women	24	49
Program	Video	App	113	149	Video	App	15	58
Techn. affinity	Low	High	129	133	Low	High	29	44
Job	-	-	-	-	Only tele-therapist	On-site and teletherapist	15	58

Table S3b. Results of Shapiro-Wilk- and Levene-Tests and corresponding selection of the statistical test regarding the training need of competency indices by groups

		**Patients**			**Therapists**		
Competency	Group Variable	Normal Distri-bution	Equal variances	Statistical test	Normal Distribu-tion	Equal variances	Statistical test
Knowledge Index							
	Age	Yes	Yes	T-Test	Yes	Yes	T-Test
	Gender	Yes	Yes	T-Test	Yes	Yes	T-Test
	Program	No	Yes	T-Test*	Yes	Yes	T-Test
	Tech. Affinity	Yes	Yes	T-Test	Yes	No	Welch-test
	Job	X	X	X	Yes	Yes	T-Test
Skill Index							
	Age	No	Yes	T-Test*	No	Yes	T-Test*
	Gender	No	Yes	T-Test*	No	Yes	U-test**
	Program	No	Yes	T-Test*	No	Yes	U-test**
	Tech. Affinity	Yes	No	Welch-test	No	Yes	U-test**
	Job	X	X	X	No	Yes	U-test**
Attitude Index							
	Age	No	Yes	T-Test*	Yes	Yes	T-Test
	Gender	No	Yes	T-Test*	Yes	Yes	T-Test
	Program	No	Yes	T-Test*	Yes	Yes	T-Test
	Tech. Affinity	No	Yes	T-Test*	Yes	Yes	T-Test
	Job	X	X	X	Yes	Yes	T-Test
Experience Index							
	Age	No	Yes	T-Test*	No	Yes	T-Test*
	Gender	No	Yes	T-Test*	No	Yes	U-test**
	Program	No	Yes	T-Test*	No	Yes	U-test**
	Tech. Affinity	No	Yes	T-Test*	No	Yes	U-test**
	Job	X	X	X	No	Yes	U-test**

* T-test applicable because sizes of group 1 and 2 > 30
** T-test not applicable, because sizes of group 1 and/or 2 <=30
level of significance p < 0,05

Table S3c. Results of Shapiro-Wilk- and Levene-Tests and corresponding selection of the statistical test regarding the training need of competencies by groups (Part 1)

		Patients			Therapists		
Competency	Group Variable	Normal Distributed	Equal variances	Statistical test	Normal Distributed	Equal variances	Statistical test
Telerehab. Knowlege							
	Age	Yes	Yes	T-Test	No	Yes	T-Test*
	Gender	Yes	Yes	T-Test	No	Yes	U-test**
	Program	Yes	Yes	T-Test	No	Yes	U-test**
	Tech.Affinity	No	No	U-test	No	Yes	U-test**
	Job	X	X	X	No	Yes	U-test**
Legal Knowledge							
	Age	No	Yes	T-Test*	Yes	Yes	T-Test
	Gender	No	Yes	T-Test*	No	Yes	U-test**
	Program	No	Yes	T-Test*	Yes	Yes	T-Test
	Tech.Affinity	No	Yes	T-Test*	Yes	No	Welch-test
	Job	X	X	X	Yes	Yes	T-Test
Technology Knowledge							
	Age	No	Yes	T-Test*	Yes	Yes	T-Test
	Gender	No	No	U-test	Yes	Yes	T-Test
	Program	No	Yes	T-Test*	Yes	Yes	T-Test
	Tech.Affinity	Yes	No	Welch-test	Yes	Yes	T-Test
	Job	X	X	X	Yes	Yes	T-Test
Medical Knowledge							
	Age	Yes	Yes	T-Test	No	No	U-test
	Gender	Yes	Yes	T-Test	Yes	Yes	T-Test
	Program	Yes	No	Welch-test	No	Yes	U-test**
	Tech.Affinity	No	No	U-test	Yes	Yes	T-Test
	Job	X	X	X	Yes	Yes	T-Test
Implement. Knowledge							
	Age	X	X	X	Yes	Yes	T-Test
	Gender	X	X	X	Yes	Yes	T-Test
	Program	X	X	X	Yes	Yes	T-Test
	Tech.Affinity	X	X	X	Yes	No	Welch-test
	Job	X	X	X	Yes	Yes	T-Test
Process Knowledge							
	Age	X	X	X	Yes	Yes	T-Test
	Gender	X	X	X	Yes	Yes	T-Test
	Program	X	X	X	Yes	Yes	T-Test
	Tech.Affinity	X	X	X	No	Yes	U-test**
	Job	X	X	X	Yes	Yes	T-Test
Technology Skills							
	Age	No	Yes	T-Test*	Yes	No	Welch-test
	Gender	No	Yes	T-Test*	No	No	U-test
	Program	No	Yes	T-Test*	No	No	U-test
	Tech.Affinity	No	No	U-test	No	Yes	U-test**
	Job	X	X	X	Yes	Yes	T-Test

* T-test applicable, because sizes of group 1 and 2 > 30

** T-test not applicable, because sizes of group 1 and/or 2 <=30

Table S3c. Results of Shapiro-Wilk- and Levene-Tests and corresponding selection of the statistical test regarding the training need of competencies by groups (Part 2)

		**Patients**			**Therapists**		
Competency	Group Variable	Normal Distributed	Equal variances	Statistical test	Normal Distributed	Equal variances	Statistical test
Adaptability							
	Age	No	Yes	T-Test*	No	Yes	T-Test*
	Gender	No	No	U-test	Yes	Yes	T-Test
	Program	No	Yes	T-Test*	No	Yes	U-test**
	Tech.Affinity	No	No	U-test	No	Yes	U-test**
	Job	X	X	X	No	Yes	U-test**
Reflectivity							
	Age	No	Yes	T-Test*	No	Yes	T-Test*
	Gender	No	Yes	T-Test*	No	Yes	U-test**
	Program	No	Yes	T-Test*	No	Yes	U-test**
	Tech.Affinity	No	Yes	T-Test*	No	Yes	U-test**
	Job	X	X	X	No	Yes	U-test**
Analytic Skills							
	Age	Yes	Yes	T-Test	Yes	Yes	T-Test
	Gender	Yes	Yes	T-Test	Yes	Yes	T-Test
	Program	No	Yes	T-Test*	Yes	Yes	T-Test
	Tech.Affinity	Yes	No	Welch-test	Yes	Yes	T-Test
	Job	X	X	X	No	Yes	U-test**
Empathic Capacity							
	Age	No	Yes	T-Test*	No	Yes	T-Test*
	Gender	No	Yes	T-Test*	No	Yes	U-test**
	Program	No	No	U-test	No	No	U-test
	Tech.Affinity	No	Yes	T-Test*	No	No	U-test
	Job	X	X	X	No	Yes	U-test**
Teamwork Skills							
	Age	No	Yes	T-Test*	No	Yes	T-Test*
	Gender	No	Yes	T-Test*	No	Yes	U-test**
	Program	No	No	U-test	No	Yes	U-test**
	Tech.Affinity	No	Yes	T-Test*	No	Yes	U-test**
	Job	X	X	X	No	Yes	U-test**
Communic. Skills							
	Age	No	Yes	T-Test*	No	Yes	T-Test*
	Gender	No	Yes	T-Test*	No	Yes	U-test**
	Program	No	Yes	T-Test*	No	Yes	U-test**
	Tech.Affinity	No	No	U-test	No	Yes	U-test**
	Job	X	X	X	No	Yes	U-test**
Motivational Skills							
	Age	No	Yes	T-Test*	No	Yes	T-Test*
	Gender	No	Yes	T-Test*	No	Yes	U-test**
	Program	Yes	No	Welch-test	No	Yes	U-test**
	Tech.Affinity	No	Yes	T-Test*	No	Yes	U-test**
	Job	X	X	X	No	Yes	U-test**

* T-test applicable, because sizes of group 1 and 2 > 30

** T-test not applicable, because sizes of group 1 and/or 2 <=30

Table S3c. Results of Shapiro-Wilk- and Levene-Tests and corresponding selection of the statistical test regarding the training need of competencies by groups (Part 3)

		Patients			Therapists		
Competency	Group Variable	Normal Distributed	Equal variances	Statistical test	Normal Distributed	Equal variances	Statistical test
Self-Management							
	Age	No	Yes	T-Test*	No	Yes	T-Test*
	Gender	No	Yes	T-Test*	No	Yes	U-test**
	Program	No	No	U-test	No	Yes	U-test**
	Tech.Affinity	No	No	U-test	No	Yes	U-test**
	Job	X	X	X	No	Yes	U-test**
Patience							
	Age	No	Yes	T-Test*	No	No	U-test
	Gender	No	Yes	T-Test*	No	Yes	U-test**
	Program	No	Yes	T-Test*	Yes	No	Welch-test
	Tech.Affinity	Yes	Yes	T-Test	Yes	Yes	T-Test
	Job	X	X	X	No	Yes	U-test**
Self-awareness							
	Age	No	Yes	T-Test*	No	Yes	T-Test*
	Gender	Yes	Yes	T-Test	No	Yes	U-test**
	Program	Yes	Yes	T-Test	No	Yes	U-test**
	Tech.Affinity	No	No	U-test	No	Yes	U-test**
	Job	X	X	X	No	Yes	U-test**
Reading/writing Skills							
	Age	No	Yes	T-Test*	X	X	X
	Gender	No	Yes	T-Test*	X	X	X
	Program	No	No	U-test	X	X	X
	Tech.Affinity	No	Yes	T-Test*	X	X	X
	Job	X	X	X	X	X	X
Therapeutic-professional Skills							
	Age	X	X	X	No	No	U-test
	Gender	X	X	X	No	No	U-test
	Program	X	X	X	No	Yes	U-test**
	Tech.Affinity	X	X	X	No	Yes	U-test**
	Job	X	X	X	Yes	Yes	T-Test
Technology Affinity							
	Age	No	Yes	T-Test*	Yes	Yes	T-Test
	Gender	Yes	Yes	T-Test	Yes	Yes	T-Test
	Program	Yes	Yes	T-Test	Yes	Yes	T-Test
	Tech.Affinity	Yes	Yes	T-Test	Yes	Yes	T-Test
	Job	X	X	X	Yes	Yes	T-Test
Technology Acceptance							
	Age	No	Yes	T-Test*	Yes	Yes	T-Test
	Gender	No	Yes	T-Test*	Yes	Yes	T-Test
	Program	No	Yes	T-Test*	Yes	Yes	T-Test
	Tech.Affinity	No	Yes	T-Test*	Yes	Yes	T-Test
	Job	X	X	X	No	Yes	U-test**

* T-test applicable, because sizes of group 1 and 2 > 30

** T-test not applicable, because sizes of group 1 and/or 2 <=30

Table S3c. Results of Shapiro-Wilk- and Levene-Tests and corresponding selection of the statistical test regarding the training need of competencies by groups (Part 4)

		**Patients**			**Therapists**		
Competency	Group Variable	Normal Distributed	Equal variances	Statistical test	Normal Distributed	Equal variances	Statistical test
Willingness to learn							
	Age	No	Yes	T-Test*	Yes	Yes	T-Test
	Gender	No	Yes	T-Test*	Yes	Yes	T-Test
	Program	No	Yes	T-Test*	Yes	Yes	T-Test
	Tech.Affinity	No	Yes	T-Test*	Yes	Yes	T-Test
	Job	X	X	X	Yes	Yes	T-Test
Open-mindedness							
	Age	No	Yes	T-Test*	No	Yes	T-Test*
	Gender	No	Yes	T-Test*	No	Yes	U-test**
	Program	No	Yes	T-Test*	No	Yes	U-test**
	Tech.Affinity	No	Yes	T-Test*	No	Yes	U-test**
	Job	X	X	X	No	Yes	U-test**
Frustrat. tolerance							
	Age	No	Yes	T-Test*	Yes	Yes	T-Test
	Gender	No	Yes	T-Test*	No	Yes	U-test**
	Program	No	Yes	T-Test*	No	Yes	U-test**
	Tech.Affinity	No	Yes	T-Test*	Yes	Yes	T-Test
	Job	X	X	X	Yes	Yes	T-Test
Self-efficacy expectation							
	Age	No	Yes	T-Test*	Yes	Yes	T-Test
	Gender	No	Yes	T-Test*	Yes	Yes	T-Test
	Program	No	Yes	T-Test*	Yes	Yes	T-Test
	Tech.Affinity	No	No	U-test	Yes	Yes	T-Test
	Job	X	X	X	Yes	Yes	T-Test
Self-interest in the program							
	Age	No	Yes	T-Test*	Yes	Yes	T-Test
	Gender	No	Yes	T-Test*	Yes	Yes	T-Test
	Program	No	No	U-test	Yes	Yes	T-Test
	Tech.Affinity	No	No	U-test	Yes	Yes	T-Test
	Job	X	X	X	Yes	Yes	T-Test
Experience with analoge therapy							
	Age	No	Yes	T-Test*	No	Yes	T-Test*
	Gender	No	Yes	T-Test*	No	Yes	U-test**
	Program	No	Yes	T-Test*	No	Yes	U-test**
	Tech.Affinity	No	Yes	T-Test*	No	No	U-test
	Job	X	X	X	No	Yes	U-test**

* T-test applicable, because sizes of group 1 and 2 > 30
** T-test not applicable, because sizes of group 1 and/or 2 <=30

Table S3c. Results of Shapiro-Wilk- and Levene-Tests and corresponding selection of the statistical test regarding the training need of competencies by groups (Part 5)

		Patients			Therapists		
Competency	Group Variable	Normal Distributed	Equal variances	Statistical test	Normal Distributed	Equal variances	Statistical test
Experience with digital health / work apps							
	Age	No	Yes	T-Test*	Yes	Yes	T-Test
	Gender	No	Yes	T-Test*	Yes	Yes	T-Test
	Program	No	No	U-test	Yes	Yes	T-Test
	Tech.Affinity	No	Yes	T-Test*	No	Yes	U-test**
	Job	X	X	X	Yes	Yes	T-Test
Experience with digital tools							
	Age	No	Yes	T-Test*	No	Yes	T-Test*
	Gender	No	Yes	T-Test*	No	Yes	U-test**
	Program	No	Yes	T-Test*	No	Yes	U-test**
	Tech.Affinity	No	Yes	T-Test*	No	Yes	U-test**
	Job	X	X	X	No	Yes	U-test**

* T-test applicable, because sizes of group 1 and 2 > 30

** T-test not applicable, because sizes of group 1 and/or 2 <=30
