# Supplementary material for: Training needs in telerehabilitation: results of a cross-sectional online survey with therapists and patients
Source: Front Public Health. 2025 Dec 11;13:1688055. doi: 10.3389/fpubh.2025.1688055 (PMC12738953; doi:10.3389/fpubh.2025.1688055)
Supplement: Supplementary file 4 [file Supplementary_file_4.pdf]

S4 appendix. Telerehabilitation usage

Table S4. Characteristics of telerehabilitation usage ($n_{patients}$ = 262, $n_{therapists}$=73)

		**Patients**		**Therapists**	
Variable	Value	n	%	n	%
Telerehab program					
	Independent app use by patients	149	56.9	58	79.5
	Therapist-led video conferencing	113	43.1	15	20.5
	N	262	100.0	73	100.0
Indication group*					
	Psychosomatic disease	170	64.9	33	45.2
	Orthopedics	77	29.4	38	52.1
	Cardiology	9	3.4	22	30.1
	Neurology	11	4.2	23	31.5
	Oncology	14	5.3	15	20.5
	Pulmonology	2	0.8	13	17.8
	Other	16	6.1	4	5.5
	N	299	114.1	148	202.7
Frequency of use					
	Once a month or less	9	3.4	3	4.1
	Several times a month	22	8.4	7	9.6
	Once a week	110	42.0	12	16.4
	Several times a week	86	32.8	29	39.7
	Daily	35	13.4	22	30.1
	N	262	100.0	73	100.0
Premature termination					
	Yes	10	22.2	x	x
	No	35	77.8	x	x
	N	45	100.0	x	x
Access to social support					
	Yes	114	43.5	x	x
	No	148	56.5	x	x
	N	262	100.0	x	x

* Multiple answers possible. Therefore, the total number exceeds 262 or 73.
