# Supplementary material for: Training needs in telerehabilitation: results of a cross-sectional online survey with therapists and patients
Source: Front Public Health. 2025 Dec 11;13:1688055. doi: 10.3389/fpubh.2025.1688055 (PMC12738953; doi:10.3389/fpubh.2025.1688055)
Supplement: Supplementary file 5 [file Supplementary_file_5.pdf]

S5 appendix. Telerehabilitation training offers, usage and content

Table S5a. Available training offers and usage by patients and therapists, relative frequencies

	Patients (all, n=262)				Therapists (all, n=73)			
Training offer	Offered and used	Offered, not used	Not offered, but requested	Not offered, nor necessary	Offered and used	Offered, not used	Not offered, but requested	Not offered, nor necessary
Individual consultation with therapist/doctor	82.8	4.2	7.3	5.7	X	X	X	X
On-site presentation	40.5	3.8	15.3	40.5	52.1	5.5	6.9	35.6
On-site workshop with opportunity to try out	24.8	3.4	22.1	49.6	50.7	4.1	17.8	27.4
Online presentation	19.1	6.9	16.4	57.6	47.9	8.2	8.2	35.6
Online webinar (interactive)	13.7	7.6	21.8	56.9	53.4	8.2	11.0	27.4
Information videos	45.8	8.0	16.4	29.8	60.3	13.7	8.2	17.8
Written information material	46.2	6.1	13.0	34.7	80.8	1.4	6.9	11.0
One-to-one consultation (e.g. by phone, email, chat)	60.7	13.4	12.2	13.7	69.9	9.6	8.2	12.3
Module/lecture in vocational training or university	X	X	X	X	15.1	4.1	20.6	60.3
Certified training in telemedicine/ telerehabilitation	X	X	X	X	21.9	6.9	31.5	39.7

Table S5b. Extent to which the competencies were covered in existing trainings for patients and therapists (training content)

Competency	Coverage in patients training, M (SD)	Coverage in therapist training, M (SD)
Telerehab. Knowledge	5.1 (0.11)	5.4 (0.20)
Legal Knowledge	4.5 (0.12)	4.0 (0.21)
Technology Knowledge	4.3 (0.12)	3.9 (0.18)
Medical Knowledge	4.3 (0.12)	4.0 (0.23)
Implement. Knowledge	X	4.7 (0.19)
Process Knowledge	X	3.6 (0.20)
**Knowledge Index**	**4.5 (0.10)**	**4.3 (0.15)**
Technology Skills	3.8 (0.12)	3.8 (0.20)
Adaptability	4.2 (0.11)	4.0 (0.21)
Reflectivity	4.4 (0.11)	3.6 (0.22)
Analytic Skills	4.2 (0.11)	3.8 (0.22)
Empathic Capacity	4.4 (0.11)	3.4 (0.22)
Teamwork Skills	4.2 (0.12)	3.5 (0.23)
Communic. Skills	4.6 (0.11)	3.8 (0.23)
Motivational Skills	4.7 (0.11)	3.9 (0.22)
Self-Management	4.8 (0.11)	3.8 (0.23)
Patience	4.3 (0.11)	3.2 (0.22)
Self-awareness	4.8 (0.11)	3.1 (0.21)
Reading/writing Skills	3.9 (0.13)	X
Therapeutic-prof. Skills	X	4.2 (0.22)
**Skills Index**	**4.4 (0.10)**	**3.7 (0.19)**
Technology Affinity	3.4 (0.12)	3.2 (0.21)
Technology Acceptance	3.6 (0.12)	3.7 (0.21)
Willingness to learn	4.4 (0.12)	3.7 (0.21)
Open-mindedness	4.6 (0.12)	3.9 (0.22)
Frustration tolerance	4.0 (0.11)	3.1 (0.20)
Self-efficacy expectation	4.4 (0.11)	3.5 (0.22)
Self-interest in program	5.0 (0.12)	3.9 (0.23)
**Attitude Index**	**4.2 (0.10)**	**3.6 (0.19)**
