# Supplementary material for: Training needs in telerehabilitation: results of a cross-sectional online survey with therapists and patients
Source: Front Public Health. 2025 Dec 11;13:1688055. doi: 10.3389/fpubh.2025.1688055 (PMC12738953; doi:10.3389/fpubh.2025.1688055)
Supplement: Supplementary file 6 [file Supplementary_file_6.pdf]

S6 appendix. Training needs regarding telerehabilitation competencies

Table S6a. Training needs of competencies accessed by patients and therapists

	**Patients (all, n=262)**			**Therapists (all, n=73)**		
Competency	Relevance, M (SD)	Performance, M (SD)	Training need, M (SD)	Relevance, M (SD)	Performance, M (SD)	Training need, M (SD)
Telerehab. Knowledge	5.3 (0.10)	5.2 (0.09)	-0.17 (0.11)	6.1 (0.19)	5.6 (0.17)	-0.41 (0.16)
Legal Knowledge	5.0 (0.12)	4.8 (0.11)	-0.24 (0.12)	5.1 (0.19)	4.8 (0.20)	-0.32 (0.17)
Technology Knowledge	5.2 (0.10)	4.7 (0.11)	-0.44 (0.12)	4.7 (0.19)	4.4 (0.22)	-0.33 (0.19)
Medical Knowledge	5.4 (0.11)	4.9 (0.09)	-0.48 (0.11)	6.1 (0.17)	6.1 (0.16)	-0.04 (0.12)
Implemention Knowledge	X	X	X	5.4 (0.19)	5.0 (0.20)	-0.32 (0.17)
Process Knowledge	X	X	X	4.4 (0.17)	4.4 (0.19)	0.01 (0.16)
**Knowledge Index**	**5.2 (0.08)**	**4.9 (0.07)**	**-0.33 (0.09)**	**5.3 (0.13)**	**5.1 (0.15)**	**-0.23 (0.11)**
Technology Skills	5.2 (0.09)	5.0 (0.10)	-0.18 (0.12)	4.6 (0.22)	4.8 (0.21)	0.14 (0.16)
Adaptability	5.4 (0.08)	5.1 (0.09)	-0.31 (0.09)	5.7 (0.18)	5.9 (0.17)	0.15 (0.12)
Reflectivity	5.7 (0.08)	5.4 (0.08)	-0.35 (0.09)	5.5 (0.18)	5.8 (0.17)	0.29 (0.12)
Analytic Skills	5.4 (0.10)	5.1 (0.09)	-0.24 (0.10)	5.5 (0.18)	5.8 (0.16)	0.30 (0.11)
Empathic Capacity	5.4 (0.11)	5.5 (0.09)	0.18 (0.10)	5.9 (0.19)	6.1 (0.17)	0.22 (0.12)
Teamwork Skills	5.3 (0.11)	5.5 (0.09)	0.18 (0.10)	4.6 (0.23)	5.9 (0.19)	1.29 (0.20)
Communic. Skills	5.7 (0.10)	5.6 (0.08)	-0.15 (0.10)	5.8 (0.19)	5.9 (0.17)	0.07 (0.15)
Motivational Skills	5.7 (0.09)	5.1 (0.09)	-0.69 (0.10)	5.8 (0.19)	5.7 (0.18)	-0.07 (0.16)
Self-Management	5.8 (0.08)	5.5 (0.09)	-0.33 (0.10)	5.8 (0.19)	6.1 (0.17)	0.36 (0.13)
Patience	5.5 (0.10)	4.6 (0.09)	-0.94 (0.12)	5.3 (0.19)	5.5 (0.17)	0.18 (0.13)
Self-awareness	5.9 (0.08)	5.1 (0.09)	-0.78 (0.10)	5.2 (0.21)	5.6 (016)	0.48 (0.16)
Reading/writing Skills	5.1 (0.11)	6.3 (0.07)	1.19 (0.12)	X	X	X
Therapeutic-prof. Skills	X	X	X	6.3 (0.17)	6.2 (0.17)	-0.15 (0.08)
**Skills Index**	**5.5 (0.07)**	**5.3 (0.06)**	**-0.20 (0.07)**	**5.5 (0.15)**	**5.8 (0.15)**	**0.27 (0.08)**
Technology Affinity	4.7 (0.09)	4.9 (0.10)	0.18 (0.11)	4.5 (0.19)	4.8 (0.19)	0.30 (0.16)
Technology Acceptance	5.3 (0.09)	5.3 (0.09)	-0.01 (0.09)	5.6 (0.18)	5.4 (0.19)	-0.19 (0.13)
Willingness to learn	5.7 (0.08)	5.8 (0.07)	0.08 (0.07)	5.7 (0.17)	5.9 (0.17)	0.12 (0.10)
Open-mindedness	5.8 (0.08)	5.8 (0.07)	0.03 (0.08)	6.0 (0.17)	5.9 (0.17)	-0.05 (0.08)
Frustration tolerance	5.2 (0.09)	4.7 (0.09)	-0.48 (0.10)	5.1 (0.17)	5.1 (0.18)	-0.04 (0.16)
Self-efficacy expectation	5.3 (0.08)	5.0 (0.08)	-0.32 (0.09)	5.4 (0.17)	5.5 (0.17)	0.04 (0.11)
Self-interest in the program	6.3 (0.07)	6.2 (0.07)	-0.13 (0.06)	5.2 (0.20)	5.3 (0.20)	0.12 (0.16)
**Attitude Index**	**5.5 (0.06)**	**5.4 (0.06)**	**-0.09 (0.06)**	**5.4 (0.15)**	**5.4 (0.15)**	**0.04 (0.08)**
Experience analoge therapy	3.9 (0.13)	4.5 (0.13)	0.58 (0.12)	5.6 (0.21)	5.9 (0.18)	0.26 (0.17)
Experience health/work apps	3.5 (0.12)	3.7 (0.13)	0.15 (0.12)	4.3 (0.20)	5.1 (0.19)	0.81 (0.19)
Experience digital tools	5.0 (0.10)	5.4 (0.10)	0.35 (0.10)	5.2 (0.18)	5.7 (0.18)	0.56 (0.15)
**Experience Index**	**4.2 (0.09)**	**4.5 (0.09)**	**0.36 (0.09)**	**5.0 (0.17)**	**5.6 (0.16)**	**0.54 (0.14)**

Table S6b. Training needs of competencies by patient and therapist subgroups

	**Patients (video user, n=113)**	**Patients (app user, n=149)**	**Therapists (video user, n=15)**	**Therapists (app user, n=58)**	**Therapists (tele, n=15)**	**Therapists (on-site, n=58)**
Competency	Training need, M (SD)	Training need, M (SD)	Training need, M (SD)	Training need, M (SD)	Training need, M (SD)	Training need, M (SD)
Telerehab. Knowledge	-0.12 (0.17)	-0.21 (0.14)	-0.40 (0.39)	-0.41 (0.18)	-0.13 (0.41)	-0.48 (0.17)
Legal Knowledge	-0.29 (0.18)	-0.19 (0.15)	-0.13 (0.34)	-0.36 (0.19)	-0.20 (0.43)	-0.34 (0.18)
Technology Knowledge	-0.30 (0.19)	-0.55 (0.14)	-0.60 (0.53)	-0.26 (0.20)	-0.13 (0.42)	-0.38 (0.22)
Medical Knowledge	-0.40 (0.18)	-0.54 (0.13)	-0.20 (0.14)	0.00 (0.15)	-0.07 (0.27)	-0.03 (0.13)
Implemention Knowledge	X	X	-0.27 (0.46)	-0.33 (0.18)	-0.07 (0.42)	-0.38 (0.19)
Process Knowledge	X	X	-0.27 (0.23)	0.09 (0.20)	0.47 (0.40)	-0.10 (0.18)
**Knowledge Index**	**-0.28 (0.14)**	**-0.37 (0.10)**	**-0.31 (0.29)**	**-0.21 (0.12)**	**-0.02 (0.23)**	**-0.29 (0.12)**
Technology Skills	-0.08 (0.18)	-0.26 (0.16)	-0.13 (0.50)	0.21 (0.15)	0.07 (0.28)	0.16 (0.18)
Adaptability	-0.33 (0.14)	-0.30 (0.11)	-0.33 (0.25)	0.28 (0.14)	0.40 (0.31)	0.09 (0.14)
Reflectivity	-0.41 (0.14)	-0.30 (0.12)	0.27 (0.25)	0.29 (0.14)	0.20 (0.22)	0.31 (0.14)
Analytic Skills	-0.36 (0.15)	-0.15 (0.13)	0.20 (0.26)	0.33 (0.12)	0.47 (0.17)	0.26 (0.13)
Empathic Capacity	-0.04 (0.13)	0.34 (0.15)	0.07 (0.15)	0.26 (0.15)	-0.20 (0.14)	0.33 (0.14)
Teamwork Skills	-0.19 (0.14)	0.46 (0.14)	1.07 (0.40)	1.34 (0.24)	1.27 (0.50)	1.29 (0.22)
Communic. Skills	-0.35 (0.12)	0.01 (0.14)	-0.27 (0.18)	0.16 (0.19)	-0.47 (0.19)	0.21 (0.18)
Motivational Skills	-0.62 (0.17)	-0.74 (0.11)	-0.27 (0.23)	-0.02 (0.19)	-0.47 (0.26)	0.03 (0.19)
Self-Management	-0.16 (0.18)	-0.46 (0.10)	0.60 (0.39)	0.29 (0.13)	0.47 (0.24)	0.33 (0.15)
Patience	-0.93 (0.18)	-0.95 (0.16)	0.00 (0.10)	0.22 (0.16)	0.47 (0.32)	0.10 (0.13)
Self-awareness	-0.97 (0.16)	-0.64 (0.13)	0.27 (0.34)	0.53 (0.18)	0.47 (0.32)	0.48 (0.18)
Reading/writing Skills	1.68 (0.20)	0.81 (0.14)	X	X	X	X
Therapeutic-prof. Skills	X	X	-0.33 (0.16)	-0.10 (0.09)	-0.13 (0.19)	-0.16 (0.09)
**Skills Index**	**-0.23 (0.11)**	**-0.18 (0.09)**	**0.09 (0.14)**	**0.32 (0.09)**	**0.21 (0.14)**	**0.29 (0.10)**
Technology Affinity	0.47 (0.16)	-0.04 (0.16)	0.13 (0.47)	0.34 (0.17)	1.13 (0.29)	0.09 (0.18)
Technology Acceptance	0.15 (0.13)	-0.13 (0.13)	-0.13 (0.36)	-0.21 (0.13)	-0.33 (0.19)	-0.16 (0.15)
Willingness to learn	0.35 (0.11)	-0.12 (0.10)	0.40 (0.21)	0.05 (0.11)	0.07 (0.15)	0.14 (0.12)
Open-mindedness	0.22 (0.11)	-0.11 (0.11)	0.00 (0.20)	-0.07 (0.09)	0.07 (0.12)	-0.09 (0.10)
Frustration tolerance	-0.42 (0.16)	-0.52 (0.14)	-0.67 (0.30)	0.12 (0.19)	0.53 (0.31)	-0.19 (0.19)
Self-efficacy expectation	-0.33 (0.14)	-0.32 (0.12)	0.13 (0.24)	0.02 (0.13)	0.13 (0.29)	0.02 (0.12)
Self-interest in the program	0.03 (0.08)	-0.26 (0.09)	0.00 (0.47)	0.16 (0.17)	0.13 (0.24)	0.12 (0.20)
**Attitude Index**	**0.07 (0.09)**	**-0.21 (0.09)**	**-0.02 (0.22)**	**0.06 (0.08)**	**0.25 (0.13)**	**-0.01 (0.09)**
Experience analoge therapy	0.93 (0.19)	0.32 (0.16)	0.33 (0.40)	0.24 (0.19)	-0.33 (0.29)	0.41 (0.20)
Experience health/work apps	0.38 (0.19)	-0.02 (0.14)	0.27 (0.45)	0.95 (0.21)	1.00 (0.35)	0.76 (0.23)
Experience digital tools	0.57 (0.14)	0.19 (0.13)	0.33 (0.35)	0.62 (0.16)	0.67 (0.32)	0.53 (0.17)
**Experience Index**	**0.63 (0.14)**	**0.16 (0.11)**	**0.31 (0.35)**	**0.60 (0.15)**	**0.44 (0.25)**	**0.57 (0.16)**
