# Supplementary material for: Training needs in telerehabilitation: results of a cross-sectional online survey with therapists and patients
Source: Front Public Health. 2025 Dec 11;13:1688055. doi: 10.3389/fpubh.2025.1688055 (PMC12738953; doi:10.3389/fpubh.2025.1688055)
Supplement: Supplementary file 7 [file Supplementary_file_7.pdf]

S7 appendix. Results of T-Tests and U-Tests

Table S7a. Results of T-Tests and U-Tests regarding the training needs of competency indices

		Patients		Therapists	
Competency	Group variable	Test	P-value	Test	P-value
Knowledge index					
	Age	T-Test	0.0823	T-Test	0.9370
	Gender	T-Test	0.0089*	T-Test	0.0370*
	Program	T-Test	0.5732	T-Test	0.7177
	Technology affinity	T-Test	0.0005*	Welch-test	0.0018*
	Job	x		T-Test	0.3289
Skill index					
	Age	T-Test	0.1563	T-Test	0.9473
	Gender	T-Test	0.8229	U-test	0.0891
	Program	T-Test	0.7310	U-test	0.2340
	Technology affinity	Welch-test	0.0868	U-test	0.2356
	Job	x	x	U-test	0.6418
Attitude index					
	Age	T-Test	0.4510	T-Test	0.7477
	Gender	T-Test	0.0046*	T-Test	0.0422*
	Program	T-Test	0.0285*	T-Test	0.6876
	Technology affinity	T-Test	0.0000*	T-Test	0.0269*
	Job	x	x	T-Test	0.1828
Experience Index					
	Age	T-Test	0.1529	T-Test	0.1133
	Gender	T-Test	0.6184	U-test	0.3305
	Program	T-Test	0.0092*[1]	U-test	0.1460
	Technology affinity	T-Test	0.0012*[1]	U-test	0.0177*[1]
	Job	x	x	U-test	0.7839

* significant result

[1] significant differences between groups, but both groups have no need for training

Table S7b. Results of T-Tests and U-Tests regarding the training needs of individual competencies (Part 1)

		**Patients**		**Therapists**	
Competency	Group Variable	Statistical Test	p-value	Statistical Test	p-value
Telerehab. Knowledge					
	Age	T-Test	0.2045	T-Test	0.1455
	Gender	T-Test	0.5348	U-test	0.8644
	Program	T-Test	0.6676	U-test	0.9648
	Techn. Affinity	U-test	0.0720	U-test	0.0236*
	Job	X	X	U-test	0.9297
Legal Knowledge					
	Age	T-Test	0.3175	T-Test	0.2500
	Gender	T-Test	0.0332*	U-test	0.1817
	Program	T-Test	0.6805	T-Test	0.5825
	Techn. Affinity	T-Test	0.0240*	Welch-test	0.1275
	Job	X	X	T-Test	0.7279
Technology Knowledge					
	Age	T-Test	0.1236	T-Test	0.2922
	Gender	U-test	0.0000*	T-Test	0.0522
	Program	T-Test	0.2941	T-Test	0.4813
	Techn. Affinity	Welch-test	0.0000*	T-Test	0.0108*
	Job	X	X	T-Test	0.6121
Medical Knowledge					
	Age	T-Test	0.1298	U-test	0.3455
	Gender	T-Test	0.3187	T-Test	0.8115
	Program	Welch-test	0.5107	U-test	0.6537
	Techn. Affinity	U-test	0.1063	T-Test	0.0203*
	Job	X	X	T-Test	0.9141
Implement. Knowledge					
	Age	X	X	T-Test	0.3481
	Gender	X	X	T-Test	0.0752
	Program	X	X	T-Test	0.8882
	Techn. Affinity	X	X	Welch-test	0.0135*
	Job	X	X	T-Test	0.4698
Process Knowledge					
	Age	X	X	T-Test	0.4530
	Gender	X	X	T-Test	0.0139*
	Program	X	X	T-Test	0.3877
	Techn. Affinity	X	X	U-test	0.0244*
	Job	X	X	T-Test	0.1611
Technology Skills					
	Age	T-Test	0.1971	Welch-test	0.7061
	Gender	T-Test	0.0093*	U-test	0.0333*
	Program	T-Test	0.4498	U-test	0.8328
	Techn. Affinity	U-test	0.0000*	U-test	0.0001*
	Job	X	X	T-Test	0.8196

* significant result

[1] significant differences between groups, but both groups have no need for training

Table S7b. Results of T-Tests and U-Tests regarding the training needs of individual competencies (Part 2)

		Patients		Therapists	
Competency	Group Variable	Statistical Test	p-value	Statistical Test	p-value
Adaptability					
	Age	T-Test	0.4576	T-Test	0.4153
	Gender	U-test	0.3668	T-Test	0.3075
	Program	T-Test	0.8578	U-test	0.0584
	Techn. Affinity	U-test	0.0015*	U-test	0.2686
	Job	X	X	U-test	0.2201
Reflectivity					
	Age	T-Test	0.3703	T-Test	0.5103
	Gender	T-Test	0.0796	U-test	0.2491
	Program	T-Test	0.5618	U-test	0.9880
	Techn. Affinity	T-Test	0.6826	U-test	0.2255
	Job	X	X	U-test	0.7519
Analytic Skills					
	Age	T-Test	0.4338	T-Test	0.7238
	Gender	T-Test	0.7106	T-Test	0.0041*[1]
	Program	T-Test	0.3029	T-Test	0.6471
	Techn. Affinity	Welch-test	0.2104	T-Test	0.0272*[1]
	Job	X	X	U-test	0.2285
Empathic Capacity					
	Age	T-Test	0.3292	T-Test	0.4078
	Gender	T-Test	0.0017*	U-test	0.5244
	Program	U-test	0.3681	U-test	0.7956
	Techn. Affinity	T-Test	0.2898	U-test	0.4860
	Job	X	X	U-test	0.0698
Teamwork Skills					
	Age	T-Test	0.2618	T-Test	0.7823
	Gender	T-Test	0.3586	U-test	0.1593
	Program	U-test	0.0377*	U-test	0.6619
	Techn. Affinity	T-Test	0.9181	U-test	0.7443
	Job	X	X	U-test	0.7138
Communic. Skills					
	Age	T-Test	0.7634	T-Test	0.3361
	Gender	T-Test	0.8711	U-test	0.7142
	Program	T-Test	0.0675	U-test	0.4110
	Techn. Affinity	U-test	0.4168	U-test	0.3729
	Job	X	X	U-test	0.0467*
Motivational Skills					
	Age	T-Test	0.5999	T-Test	0.7842
	Gender	T-Test	0.6711	U-test	0.2687
	Program	Welch-test	0.5592	U-test	0.7043
	Techn. Affinity	T-Test	0.1716	U-test	0.5100
	Job	X	X	U-test	0.0465*
Self-Management					
	Age	T-Test	0.2705	T-Test	0.9102
	Gender	T-Test	0.9692	U-test	0.1011
	Program	U-test	0.0752	U-test	0.7622
	Techn. Affinity	U-test	0.0434*	U-test	0.9154
	Job	X	X	U-test	0.5603

* significant result

[1] significant differences between groups, but both groups have no need for training

Table S7b. Results of T-Tests and U-Tests regarding the training needs of individual competencies (Part 3)

		**Patients**		**Therapists**	
Competency	Group Variable	Statistical Test	p-value	Statistical Test	p-value
Patience					
	Age	T-Test	0.0107*	U-test	0.3687
	Gender	T-Test	0.8662	U-test	0.3904
	Program	T-Test	0.9428	Welch-test	0.2265
	Techn. Affinity	T-Test	0.5657	T-Test	0.3562
	Job	X	X	U-test	0.1219
Self-awareness					
	Age	T-Test	0.2413	T-Test	0.3742
	Gender	T-Test	0.5075	U-test	0.0666
	Program	T-Test	0.1004	U-test	0.3987
	Techn. Affinity	U-test	0.4444	U-test	0.6898
	Job	X	X	U-test	0.9633
Reading/writing Skills					
	Age	T-Test	0.5625	X	X
	Gender	T-Test	0.9638	X	X
	Program	U-test	0.0002*[1]	X	X
	Techn. Affinity	T-Test	0.2396	X	X
	Job	X	X	X	X
Therapeutic-professional Skills					
	Age	X	X	U-test	0.2293
	Gender	X	X	U-test	0.2889
	Program	X	X	U-test	0.3028
	Techn. Affinity	X	X	U-test	0.2442
	Job	X	X	T-Test	0.9152
Technology Affinity					
	Age	T-Test	0.7946	T-Test	0.1530
	Gender	T-Test	0.0000*	T-Test	0.0001*
	Program	T-Test	0.0263*	T-Test	0.6006
	Techn. Affinity	T-Test	0.0000*	T-Test	0.0016*
	Job	X	X	T-Test	0.0079*[1]
Technology Acceptance					
	Age	T-Test	0.5504	T-Test	0.4226
	Gender	T-Test	0.0000*	T-Test	0.1271
	Program	T-Test	0.1440	T-Test	0.8153
	Techn. Affinity	T-Test	0.0000*	T-Test	0.1536
	Job	X	X	U-test	0.3387
Willingness to learn					
	Age	T-Test	0.5325	T-Test	0.2363
	Gender	T-Test	0.9971	T-Test	0.3758
	Program	T-Test	0.0018*	T-Test	0.1581
	Techn. Affinity	T-Test	0.0089*	T-Test	0.1169
	Job	X	X	T-Test	0.7742

* significant result

[1] significant differences between groups, but both groups have no need for training

Table S7b. Results of T-Tests and U-Tests regarding the training needs of individual competencies (Part 4)

		Patients		Therapists	
Competency	Group Variable	Statistical Test	p-value	Statistical Test	p-value
Open-mindedness					
	Age	T-Test	0.3324	T-Test	0.7560
	Gender	T-Test	0.6403	U-test	0.5288
	Program	T-Test	0.0346*	U-test	0.5771
	Techn. Affinity	T-Test	0.0025*	U-test	0.9452
	Job	X	X	U-test	0.5216
Frustrat. tolerance					
	Age	T-Test	0.0822	T-Test	0.7971
	Gender	T-Test	0.6741	U-test	0.7017
	Program	T-Test	0.6345	U-test	0.0284*
	Techn. Affinity	T-Test	0.0255*	T-Test	0.0442*
	Job	X	X	T-Test	0.0763
Self-efficacy expectation					
	Age	T-Test	0.8008	T-Test	0.1947
	Gender	T-Test	0.0630	T-Test	0.0738
	Program	T-Test	0.9775	T-Test	0.6850
	Techn. Affinity	U-test	0.0010*	T-Test	0.2064
	Job	X	X	T-Test	0.6850
Self-interest in the program					
	Age	T-Test	0.2201	T-Test	0.4315
	Gender	T-Test	0.5277	T-Test	0.4799
	Program	U-test	0.0014*	T-Test	0.7015
	Techn. Affinity	U-test	0.0023*	T-Test	0.3520
	Job	X	X	T-Test	0.9751
Experience in analoge therapy					
	Age	T-Test	0.5091	T-Test	0.0004*
	Gender	T-Test	0.7714	U-test	0.3612
	Program	T-Test	0.0127*[1]	U-test	0.7490
	Techn. Affinity	T-Test	0.4999	U-test	0.4422
	Job	X	X	U-test	0.1268
Experience with digital health / work apps					
	Age	T-Test	0.1119	T-Test	0.3032
	Gender	T-Test	0.9670	T-Test	0.6967
	Program	U-test	0.1274	T-Test	0.1545
	Techn. Affinity	T-Test	0.0070*	U-test	0.1240
	Job	X	X	T-Test	0.6163
Experience with digital tools					
	Age	T-Test	0.2400	T-Test	0.4266
	Gender	T-Test	0.0962	U-test	0.0385*[1]
	Program	T-Test	0.0566	U-test	0.3380
	Techn. Affinity	T-Test	0.0000*	U-test	0.0009*[1]
	Job	X	X	U-test	0.5799

* significant result

[1] significant differences between groups, but both groups have no need for training
